# Contrasting genetic trajectories of endangered and expanding red fox populations in the western U.S

**DOI:** 10.1038/s41437-022-00522-4

**Published:** 2022-03-21

**Authors:** Cate B. Quinn, Sophie Preckler-Quisquater, Jocelyn R. Akins, Patrick R. Cross, Preston B. Alden, Stevi L. Vanderzwan, John A. Stephenson, Pete J. Figura, Gregory A. Green, Tim L. Hiller, Benjamin N. Sacks

**Affiliations:** 1 Mammalian Ecology and Conservation Unit, Veterinary Genetics Laboratory, University of California, Davis, 1 Shields Avenue, Davis, CA 95616 USA; 2 Cascades Carnivore Project, 309 Oak Street, Suite 201, Hood River, OR 97301 USA; 3 Yellowstone Ecological Research Center, 4135 Valley Commons Drive Suite D, Bozeman, MT 59718 USA; 4 Grand Teton National Park, PO Drawer 170, Moose, WY 83012 USA; 5 California Department of Fish and Wildlife, 601 Locust Street, Redding, CA 96001 USA; 6 Huxley College of the Environment, Western Washington University, Bellingham, WA 98225 USA; 7 Wildlife Ecology Institute, P.O. Box 4725, Helena, MT 59604 USA; 8 Department of Population Health and Reproduction, School of Veterinary Medicine, University of California, Davis, 1 Shields Avenue, Davis, CA 95616 USA

**Keywords:** Conservation biology, Population genetics

## Abstract

As anthropogenic disturbances continue to drive habitat loss and range contractions, the maintenance of evolutionary processes will increasingly require targeting measures to the population level, even for common and widespread species. Doing so requires detailed knowledge of population genetic structure, both to identify populations of conservation need and value, as well as to evaluate suitability of potential donor populations. We conducted a range-wide analysis of the genetic structure of red foxes in the contiguous western U.S., including a federally endangered distinct population segment of the Sierra Nevada subspecies, with the objectives of contextualizing field observations of relative scarcity in the Pacific mountains and increasing abundance in the cold desert basins of the Intermountain West. Using 31 autosomal microsatellites, along with mitochondrial and Y-chromosome markers, we found that populations of the Pacific mountains were isolated from one another and genetically depauperate (e.g., estimated Ne range = 3–9). In contrast, red foxes in the Intermountain regions showed relatively high connectivity and genetic diversity. Although most Intermountain red foxes carried indigenous western matrilines (78%) and patrilines (85%), the presence of nonindigenous haplotypes at lower elevations indicated admixture with fur-farm foxes and possibly expanding midcontinent populations as well. Our findings suggest that some Pacific mountain populations could likely benefit from increased connectivity (i.e., genetic rescue) but that nonnative admixture makes expanding populations in the Intermountain basins a non-ideal source. However, our results also suggest contact between Pacific mountain and Intermountain basin populations is likely to increase regardless, warranting consideration of risks and benefits of proactive measures to mitigate against unwanted effects of Intermountain gene flow.

## Introduction

Although conservation has historically targeted the species level, modern awareness that evolution is continuous highlights that populations can also be evolutionarily distinct, sometimes possessing local adaptations or transitioning toward speciation (Moritz 2002; Roux et al. 2016). In certain cases, long-isolated populations that evolved in distinct habitats arguably warrant greater conservation attention due to their evolutionary distinctiveness than larger or more connected populations, which may harbor greater redundancy (Lesica and Allendorf 1995; Hampe and Petit 2005). In addition to their potentially greater evolutionary value, historically small and isolated populations also tend to have lower genetic diversity (Frankham 1996) and thus face an elevated risk of extirpation due to demographic and genetic stochasticity (Frankham 2015). Anthropogenic stressors, such as development and exploitation, further exacerbate extinction risk because rapid and recent declines may disproportionately increase inbreeding depression (Kyriazis et al. 2021; van der Valk et al. 2021).

As human activities continue to contribute to habitat loss and range contractions, maintaining evolutionary processes will increasingly require targeting interventions to the population level, even for common and widespread species. Deliberate translocations of small numbers of individuals from one population to another has been advocated as a conservation tool to rescue genetically depauperate populations from the most severe fitness effects of genetic erosion (Frankham 2015; Whiteley et al. 2015; Ralls et al. 2018). However, doing so without sufficient understanding of evolutionary relationships or demographic history could potentially thwart evolutionary trajectories, risk outbreeding depression, or, depending on the genetic load of donor populations, exacerbate inbreeding depression (Edmands 2007; Bell et al. 2019; Wilder et al. 2020; Kyriazis et al. 2021). Targeting conservation measures to specific populations therefore requires detailed knowledge of population structure, both to identify populations of conservation need and value as well as to assess the suitability of potential donor populations (e.g., Frankham et al. 2011).

As a species, red foxes (*Vulpes vulpes*) have the widest distribution of any non-domestic terrestrial carnivore, ranging naturally over three continents and introduced to Australia (Larivière and Pasitschniak-Arts 1996). The IUCN considers the species conservation status of “least concern” (Hoffmann and Sillero-Zubiri 2021), yet their global distribution belies variation in morphology, ecology, and demography at local levels (e.g., Gortázar et al. 2000; Szuma 2008; Devenish-Nelson et al. 2013). The population structure of red foxes is further complicated by a 20th century history of translocating red foxes (Saunders et al. 1995; Long 2003), often for fur-farming (Petersen 1914; Ashbrook 1928). Without means to discern ancestry, it can be difficult to distinguish the demographic trends of indigenous, locally adapted populations from recently translocated, genetically divergent populations (Champagnon et al. 2012). Genetic analyses that simultaneously characterize ancestry and connectivity are thus vital in determining conservation needs of specific red fox populations, as well as informing decisions on how to manage connectivity.

In the western contiguous United States (hereafter, western U.S.), naturalists and trappers from the early 20th century reported that red foxes were largely restricted to the upper montane and subalpine life zones of the major western mountain ranges (Seton 1929; Bailey 1936a, 1936b; Grinnell et al. 1937; Dalquest 1948; Hall and Kelson 1959). Although recognized as four distinct subspecies, indigenous western red foxes form a single lineage that is >20,000 years diverged from red foxes in the remainder of the continent (see Supplementary Text SI for details on taxonomy). Available information on phenotype suggests that montane members of the lineage are also smaller, have proportionally larger foot surfaces, and breed considerably later in the year, all of which have been suggested to represent local adaptations to the subalpine environment (Grinnell et al. 1937; Roest 1977; Fuhrmann 1998; Cross et al. 2018; SCAT, 2022). Following European colonization of North America, native western red foxes declined in portions of their range presumably due to a combination of unregulated harvest and poisoning associated with predator control programs (Perrine et al. 2010; Sacks et al. 2010). Despite decades of regulation or protection, native western red foxes in some areas have yet to recover the full extent of their historical range, particularly in the Pacific mountains of the Cascades and Sierra Nevada where detections remain relatively rare (Sierra Nevada Red Fox Conservation Advisory Team [SCAT] 2022; Washington Department of Fish and Wildlife 2015).

Low abundance, limited distributions, and increased research and outreach have led to conservation attention of multiple high-elevation populations in the Pacific mountains, including listing at both state and federal levels (see Supplementary Text for details on state listing status). Most recently, the U.S. Fish and Wildlife Service designated the population of the Sierra Nevada subspecies (*V. v. necator*) residing in the Sierra Nevada as a federally endangered distinct population segment (DPS; U.S. Fish and Wildlife Service 2021). The U.S. Fish and Wildlife Service has also recognized the Southern Cascade DPS of the Sierra Nevada subspecies, composed of populations in the vicinity of Lassen Peak of northern California and the Oregon Cascades, but determined the DPS not warranted for listing, largely due to an absence of data in the Oregon Cascades at that time (U.S. Fish and Wildlife Service 2015). In all Pacific mountain regions, conservation efforts have generally been hindered by uncertainty about basic population status and limiting factors (SCAT, 2022). Previous genetic assessments have considered Pacific mountain populations individually (e.g., Akins et al. 2018, Quinn et al. 2019), but only limited attempts have been made to conduct genetic analyses at a broad scale (e.g., Aubry et al. 2009) that could both highlight important commonalities as well as facilitate conservation prioritization.

Concurrent with apparent declines in the Pacific mountains, red foxes reportedly began increasing in abundance in the Intermountain West during 1960–2000s, including expansion into habitat types and elevation bands not historically attributed to the native montane subspecies. Such increases have been observed in the Snake River Plain of Idaho, low elevations of the Basin and Range ecoregions of Nevada, Utah and southeastern Oregon, and the Columbia River Plateau of Washington and northern Oregon (Fichter and Williams 1967; Mace 1970; Verts and Carraway 1998; Hoffmann et al. 1969; Kamler and Ballard 2003; Green et al. 2017). Red foxes in these cold desert basins occupy a range of vegetative zones, including river plains, shrub-steppe, arid grasslands, and agricultural fields. Both the departure from historical montane habitat and apparent rapid increase have led some to posit that red foxes in the Intermountain basins are not members of native western montane subspecies, but rather descendants of nonnative red foxes that escaped or were released from fur farms (e.g., Fichter and Williams 1967; Mace 1970), similar to feral populations documented in western Washington and California (Aubry 1984; Lewis et al. 1999). Alternatively, these lower elevation red foxes may have also originated from a continental-scale expansion from eastern or northwestern North America (hereafter referred to as midcontinent) (e.g., Hoffmann et al. 1969; Kamler and Ballard 2002; Cross et al. 2018).

The degree to which red foxes in the cold desert basins of the Intermountain West derive from downslope expansions of the native montane subspecies, descendants of fur-farm foxes, or post-glacial expansions from the midcontinent carry different implications for their management. Fur-farm stock originated primarily from wild-caught red foxes of eastern Canada and Alaska (Laut 1921), similar to the expected composition of midcontinent red foxes (Aubry et al. 2009; Black et al. 2018). In both cases, red fox lineages from northwestern and eastern North America diverged before the Last Glacial Maximum and occupy different long-term niches (Aubry et al. 2009; Statham et al. 2014). In addition, nonindigenous fur-farm stock underwent generations of selective breeding in captivity (Laut 1921; Lord et al. 2020), potentially resulting in traits that are maladaptive in wild environments (Rhymer and Simberloff 1996; Laikre et al. 2010). Previous genetic investigations have shown largely native western mitochondrial haplotypes in the expansion zone (e.g., Statham et al. 2012; Green et al. 2017), but low sampling resolution and the absence of nuclear data has limited their conclusions.

Here, we conduct the most comprehensive range-wide analysis of the contemporary genetic structure of red foxes in the western U.S to date, with the intent of contextualizing field observations of relative scarcity in the Pacific mountains and increasing abundance in the arid basins to their east. To accomplish this, we filled critical sampling gaps in the high-elevation Oregon Cascades and low-elevation interior western U.S., where few nuclear data have been available until now. Our questions were threefold. First, do red foxes show genetic signatures of isolated, remnant populations throughout the Pacific mountains, or is it a phenomenon limited to the federally protected Sierra Nevada DPS? We were specifically interested in the southern Cascades of Oregon and California, where the conservation status has been poorly defined due to a lack of data. Second, to what extent can the apparent increases in red fox abundance and distribution in the cold desert basins be explained by nonindigenous red foxes and, if applicable, do nonindigenous foxes originate from anthropogenic translocations of the past or natural expansion from the midcontinent? Finally, we sought to characterize any contact between Pacific and Intermountain populations, as novel gene flow can rapidly alter the trajectories of small, isolated populations (Hedrick et al. 2014), the consequences of which are particularly complex when populations differ in their evolutionary histories. Results lay the foundation for conservation of the native western red fox lineage generally, and future recovery planning for the endangered Sierra Nevada red fox DPS specifically.

## Methods

### Study area and samples

**Fig. 1:**
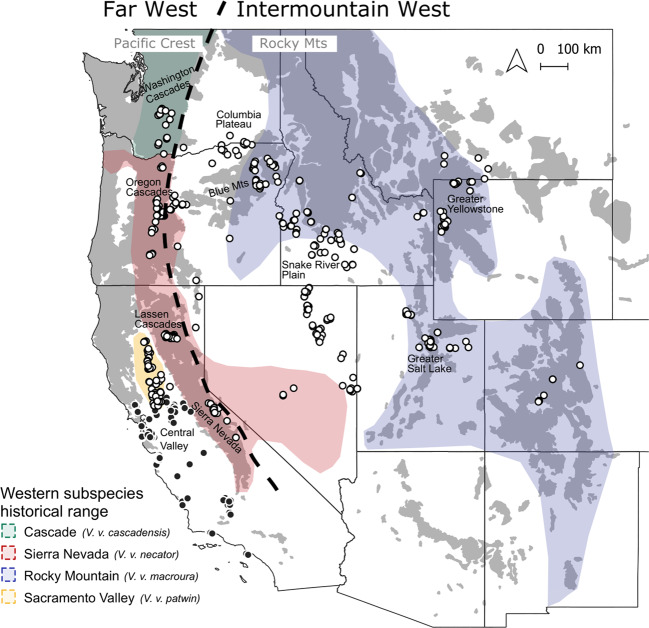
Distribution of DNA samples from red foxes (*Vulpes vulpes*; *n* = 730) in the western contiguous U.S. Circles indicate the location of collection, with dark fill specifying those known from previous studies to be introduced via fur farms (Sacks et al. 2011, 2016). Colored polygons depict coarse historical ranges of native western subspecies according to Hall and Kelson (1959) and modified by the genetic findings of Sacks et al. (2010). Finer-scaled historical habitat associations are approximated by gray shading, which depicts a merged version of Kuchler’s (1964) vegetation categories, “conifer forest” and “alpine meadows or barren”. The Far West, referred to throughout the main text, includes red foxes in the Pacific mountains (Cascades, Sierra Nevada) and westward, whereas the Intermountain West includes red foxes in the Rocky Mountains and surrounding cold desert basins (Great Basin, Columbia Plateau, Snake River Plain).

We analyzed DNA from 730 individual red foxes collected throughout the western U.S. between 1986 and 2018 (Table S1; Fig. 1). For convenience, we refer to two broad regions of the study area: (1) the “Far West,” which includes the high-elevation Pacific ranges (Cascades, Sierra Nevada) and the low-elevation valley and coastal areas to their west, and (2) the “Intermountain West,” which includes the high-elevation Rocky Mountain and Great Basin ranges and the surrounding lower elevation, cold desert basins (i.e., the Columbia Plateau, the Snake River Plain, and the lower elevations of the Great Basin). Generally, the high-elevation mountain ranges encompass the historical distribution of indigenous western red foxes (Hall and Kelson 1959), whereas lower elevations correspond to either populations known to originate from fur farms (California; Sacks et al. 2016) or those of poorly characterized ancestry (cold desert basins; Fichter and Williams 1967; Verts and Carraway 1998; Kamler and Ballard 2003). The Sacramento Valley subspecies (*V. v. patwin*) is an exception to this elevational characterization, in that it is sister to the high-elevation Sierra Nevada subspecies but occupied the grassland-dominated Sacramento Valley prior to European colonization (Sacks et al. 2010; Volkmann et al. 2015).

Most of the 730 DNA samples were used in previous studies but typed at only a subset of the genetic markers used here (e.g., mitochondrial only; see Table S1 for details); 251 samples were newly collected. The majority (62%) of samples were tissue or blood collected opportunistically from mortalities (e.g., foxes killed via vehicle strikes), pelts with permission of trappers, or live captures for other studies whose trapping and handling methods followed American Society of Mammalogists animal care guidelines (Sikes et al. 2011) and were approved by the University of California, Davis Animal Care and Use Committee (IACUC No. 17860). From more remote montane regions, we additionally incorporated noninvasively collected hair and fecal samples (e.g., Hiller et al. 2015; Akins et al. 2018). Tissue and hair were stored in desiccant or frozen at −20 °C and fecal samples were stored in >95% ethanol. We extracted DNA from feces using QIAamp Stool Kit (Qiagen Inc., Valencia CA), and from hair, urine, and tissue using DNeasy Blood and Tissue Kits (Qiagen Inc.) using previously described protocols (e.g., Quinn et al. 2019).

### Autosomal markers

We used autosomal allele frequencies to characterize genetic structure, quantify diversity, and infer demographic trends. We attempted PCR amplification of 31 microsatellite loci originally ascertained in domestic dogs but with primers revised based on red fox sequences where appropriate (Moore et al. 2010). We multiplexed loci using primers and PCR conditions previously described (e.g., Quinn et al. 2019). Amplification of each locus was attempted 1–2× for tissue and blood samples and 2–5× for hair and fecal samples, based on previously calculated allelic dropout rates of <1% for tissue- and blood-extracted DNA (Sacks et al. 2010) and <5% for fecal extracted DNA (Sacks et al. 2011; Quinn et al. 2019). For fecal and hair samples, we assigned sample genotypes to distinct individuals using previously described matching criteria (Quinn et al. 2019) and used only one genotype/individual sampled in final analyses. We excluded any final genotypes composed of <29 loci to minimize effects of missing data. Based on this dataset, we calculated per-locus heterozygosity, observed number of alleles, and degree of deviance from Hardy–Weinberg equilibrium (F_IS_) and its statistical significance using packages hierfstat (Goudet 2005) and pegas (Paradis 2010) in R v.4.0.2 (R Development Core).

#### Population structure

We used three individual-based clustering methods to explore population structure: nonspatial Bayesian clustering in Structure v. 2.3 (Pritchard et al. 2000; Falush et al. 2003), spatial Bayesian clustering in Tess v. 2.3 (Chen et al. 2007; Durand et al. 2009), and multivariate discriminant analysis of principal components (DAPC; Jombart et al. 2010). We compared results across methods, as each one has its own strengths: Structure serves as a standard, having the most widespread use; Tess accounts explicitly for spatial autocorrelation among samples and therefore should outperform other methods in the presence of isolation-by-distance (Chen et al. 2007); finally, DAPC is a multivariate method that assumes no particular underlying population processes such as Hardy–Weinberg equilibrium or linkage equilibrium (Jombart et al. 2010). In Structure, we used an admixture model with correlated allele frequencies and no prior information to run 20 simulations with the number of genetic clusters (*K*) ranging from 1 to 20 for 200,000 Markov chain Monte Carlo (MCMC) repetitions, discarding the first 100,000 as burn-in. After removing 1% of the runs with the lowest values, we calculated the mean log posterior probability of the data in Structure Harvester (Earl and vonHoldt 2012) and identified a range of reasonable *K* values based on where likelihoods approached a plateau. In Tess, we first ran ten models with no admixture for *K* = 2–20 to obtain an upper bound on the number of clusters in the data. We then plotted the deviance information criterion (DIC) for each *K* value, and ran admixture models for *K* values between two and the number of clusters for which the DIC reached a plateau in the non-admixture models. We ran 30 admixture models using the conditional autoregressive (CAR) model and used DIC to select plausible K values for comparison with other methods. For all runs we used Euclidian geographic distances for weighting, 100,000 iterations, and a 25,000 iteration burn-in period. For DAPC assignments, we used the K-means clustering algorithm and Bayesian Information Criteria in R package adegenet (Jombart 2008) to determine the number of clusters that most efficiently summarized the data. The DAPC results did not meaningfully differ when the number of principal components retained was decided using the α-score method (*n* = 14) or explanation of 80% cumulative variance (*n* = 75); we therefore present results based on retention of 75 principal components and all discriminant functions.

We tested for concordance among the three clustering algorithms and used a total evidence approach to characterize population structure (e.g., Ball et al. 2010). We chose not to average models across multiple runs (e.g., Jakobsson and Rosenberg 2007), as model averaging can falsely suggest admixture or ambiguous assignment when none exists. Instead, we visualized individual runs with the highest support (based on DIC and posterior probability) for a range of K values that seemed to describe the majority of the structure in the data (Pritchard et al. 2003). We then evaluated genetic groupings based on three criteria: stability of membership across *K* values (hierarchal stability), stability of membership across methods (model stability), and the average magnitude of *q* values (i.e., membership coefficients, admixture proportions, etc., indicative of cluster distinctiveness). We interpreted genetic clusters composed predominantly of high *q* values (e.g., >0.9) that were robust to the choice of algorithm and *K* as “discrete clusters”. In contrast, clusters displaying a broad distribution of *q* values (e.g., 0.25–0.75) that varied by model and choice of *K* suggested continuous genetic structure not easily delineated with discrete spatial boundaries (e.g., clines, admixture zones).

When isolation-by-distance contributes to population structure, the magnitude of isolation increases with distance, which can overwhelm and obscure discrete subdivisions operating locally. Due to our interest in such subdivisions, particularly the relationships between red foxes in the upper elevation zones and the more recently colonized Intermountain basins, we therefore performed additional clustering analyses at a finer spatial scale in Oregon and southeastern Washington. Within a few hundred kilometers, this region contained samples from the historical ranges of two native subspecies (Sierra Nevada red fox in the Cascades and Rocky Mountain red fox in the Blue Mountain and Wallowa ranges) as well as intermediary basins where red foxes colonized more recently (Fig. 1; Bailey 1936b; Verts and Carraway 1998). The expected variation in population histories combined with high sampling resolution suggested this region offered the greatest power to elucidate the role that montane populations played in founding the basin populations, as well as detect and assess directionality of any subsequent gene flow between low and high elevations.

#### Genetic dissimilarity

To evaluate relative degrees of genetic differentiation among clusters, we used DAPC to visualize the relationship of clusters in ordinal space. Because in this case our objective was to evaluate the genetic dissimilarity of clusters rather than designate them de novo (Miller et al. 2020), we defined the genetic groups using Tess assignments. Otherwise we conducted DAPC using the same number of PCs and discriminant functions as in the de novo cluster analysis. Next, using the same clustering results from Tess to define genetic groups (i.e., based on a maximum *q* value > 0.5), we calculated pairwise *F*_ST_ according to Weir and Cockerham (1984) in the R package hierfstat (Goudet 2005). We bootstrapped *F*_ST_ values across loci for 5000 iterations to estimate 95% confidence intervals.

To visualize spatial variation in rates of gene flow, we used the estimated effective migration surface algorithm (EEMS; Petkova et al. 2016). The EEMS approach highlights geographic regions where genetic dissimilarity decays faster or slower than expected under a strict model of isolation-by-distance. Briefly, a dense grid is overlain to assign individuals to demes, and effective migration rates are estimated among demes and adjusted so that the observed genetic dissimilarities reflect fitted values under a stepping-stone model. To ensure results were independent of grid size, we combined analyses using 500, 1000, and 1500 demes. In all cases we ran three independent MCMC chains of 1,000,000 iterations following a burn-in of 100,000 iterations, sampling every 5000 iterations. Algorithm parameters were optimized to produce the recommended 20–30% acceptance rates and we inspected log posterior plots to verify convergence. We visualized results using the R package reemsplots2 (Petkova 2020).

#### Geographic patterns of genetic diversity and effective population size

For all populations, we calculated observed (*H*_O_) and expected (*H*_E_) heterozygosities and rarefied allelic richness (*AR*) in the R package hierfstat (Goudet 2005). We estimated genetic effective population sizes (*N*_e_) using a bias-corrected version of the linkage disequilibrium method (Waples 2006; Waples and Do 2008) in the software program NeEstimator v2 (Do et al. 2014). We estimated *N*_e_ under the assumption of random mating because although red foxes have strong pair bonds, they exhibit polygyny in some circumstances (Zabel and Taggart 1989). We excluded alleles with frequencies <0.05 to balance tradeoffs of precision and bias (Waples and Do 2010) and used jackknife-based confidence intervals. Finally, while the linkage disequilibrium method has high precision when *N*_e_ is small (Waples and Do 2010), sources of linkage disequilibrium other than drift, such as gene flow and overlapping generations, can downwardly bias estimates (Waples and England 2011; Waples et al. 2014). We therefore used subsampling to explore the sensitivity of *N*_e_ estimates under different sampling schemes. We used a custom R script to randomly sample without replacement 10, 20, or 30 individuals from multiple geographic groups and then iterated estimation in NeEstimator 1000 times for each subsampling scheme and geographic group.

We delineated how individuals were aggregated for computing the above summary statistics in two ways, depending on the results of clustering analyses. For genetic clusters that were categorized as “discrete” (see “Population structure”), we drew minimum convex polygons around their members and incorporated all individuals within, including those that were assigned to different clusters and were presumed to be immigrants. For genetic clusters deemed to have more continuous or complex structure, rather than partitioning data with arbitrary geographic boundaries, we estimated summary statistics using Wright’s (1946) concept of a genetic neighborhood, which describes the local area in which most matings occur. We implemented this approach using the R package sGD (Shirk and Cushman 2011), which groups individuals into overlapping neighborhoods based on a defined radius and centered on each individual. We considered only neighborhoods with at least ten individuals and we used a radius of 85 km, based on a previous study that showed ~90% of red foxes in North Dakota disperse less than this distance (Allen and Sargeant 1993). We also tested radii of 75 and 100 km and observed no qualitative difference in results.

To visualize spatial patterns of diversity, we interpolated a continuous spatially explicit surface for each diversity and N_e_ metric using inverse distance weighting in the R package gstat (Pebesma 2004). All R scripts used to modify the sGD function and visualize genetic diversity and effective population size estimates as an interpolated surface are available at https://github.com/cbquinn/westernRFstructure.

### Uniparentally inherited markers

We primarily used uniparentally inherited markers on the mitochondrial genome and Y chromosome to investigate the contribution of nonindigenous lineages to the genetic composition of red foxes in the western U.S. The absence of recombination and their structuring at phylogeographic timescales (e.g., thousands to hundreds of thousands of years) allowed us to discriminate matrilines and patrilines indigenous to the western U.S. from those that originated in other parts of the continent (i.e., eastern and northwestern lineages; Aubry et al. 2009). Secondarily, we used uniparentally inherited markers to test the geographic patterns of diversity and structure inferred using autosomal markers. With one-quarter of the effective population sizes of autosomal markers, Y chromosomes and mitochondrial DNA can be sensitive indicators of loss of diversity through drift or founder effect (Moritz 1994).

#### Mitochondria

For each individual, we amplified a 354 bp segment of the cytochrome b gene (primers RF14724 and RF15149; Perrine et al. 2007) and a 343 bp segment of the D-loop (primers VVDL1 and VVDL6; Aubry et al. 2009). We concatenated the two fragments as “C-D,” where C indicates the name of the cytochrome *b* haplotype and D indicates the numeric name of the D-loop haplotype, according to the convention of previous studies (e.g., Sacks et al. 2010). Because of widespread use of these markers in previous studies (e.g., Aubry et al. 2009; Sacks et al. 2010; Merson et al. 2017), we could reasonably impute one of the two fragments in cases where only a single combination had been previously documented (noted in Table S1). For A-19, a widespread haplotype ancestral to all other haplotypes in the Mountain subclade (e.g., Sacks et al. 2010), we additionally amplified a 200 bp fragment of cytochrome *b* (primers VVmc-780F and VVmc-980R; Volkmann et al. 2015) containing a single SNP that resolves A-19 into two subhaplotypes (A-19a, A-19b). For all mitochondrial fragments, we used previously published PCR conditions and reagent mixtures (Perrine et al. 2007; Aubry et al. 2009; Quinn et al. 2019).

Multiple previous studies have described mitochondrial variation in red foxes in wild North American populations and globally distributed fur farms (e.g., Aubry et al. 2009; Sacks et al. 2010; Statham et al. 2011, 2012, 2014; Kasprowicz et al. 2016; Lounsberry et al. 2017; Merson et al. 2017; Black et al. 2018; Cross et al. 2018). Using all known published homologous mtDNA haplotypes sampled in wild populations in North America or fur farms, we recreated a median joining network in PopART (Leigh and Bryant 2015) and identified haplotypes as belonging to one of four previously described matrilineal groups: the Holarctic clade corresponding to Alaska and northwestern Canada, the Eastern subclade corresponding to eastern North America, the Mountain subclade corresponding to the western contiguous U.S., and the Widespread subclade that is ancestral to both the Mountain and Eastern subclades (i.e., member haplotypes occur in either eastern North America or the western U.S. but not in both; Aubry et al. 2009; Sacks et al. 2010). We conservatively assumed all haplotypes belonging to the Mountain and Widespread subclades were indigenous western matrilines, with the exceptions of two haplotypes (K-36 and O-24) previously documented in fur farms (Lounsberry et al. 2017; Black et al. 2018). Historically, the O-24 haplotype was exclusive to the Washington Cascades, so we considered it native in Washington and fur farm-derived elsewhere. For estimates of matrilineal diversity, we calculated gene diversity (the equivalent of expected heterozygosity for diploid data) according to Nei (1987). For continuous populations, we modified the sGD function to calculate gene diversity using haplotype frequency data (i.e., according to Nei 1987) with the same neighborhood approach as described for autosomal diversity.

#### Y chromosome

A Y-chromosome phylogeographic network for North American red foxes has yet to be resolved. We therefore utilized faster-mutating Y-microsatellites to assess diversity in the patriline and slower-mutating Y-SNPs to evaluate lineage introgression. First, we determined a Y-chromosome microsatellite haplotype consisting of 14 linked loci (Statham et al. 2014; Rando et al. 2017). We allowed for missing data at ≤3 loci and used linkage to impute them based on an exclusive 100% match of the 11–14 alleles present with another complete haplotype, for a total of 282 Y-microsatellite haplotypes in the western U.S. and 359 reference haplotypes sampled from non-western North American populations. The PCR protocols were identical to those for autosomal microsatellites and are described in full by Quinn et al. (2019). We then used Y-microsatellite haplotypes to estimate gene diversity for the patriline in the study area using the same combination of discrete and neighborhood approaches as for mitochondrial sequences.

Because high rates of homoplasy in Y-microsatellites can obscure a deeper phylogenetic signal (Wei et al. 2013), we used slower mutating Y-SNPs to determine the origin of the patriline. We assayed all Y-SNPs with the Sequenom MassARRAY iPLEX platform (Agena Biosciences, Inc., San Diego, California, USA) using cycling conditions described by Sacks et al. (2021). The sequences and concentrations for PCR and extension primers are presented in Table S3 and the flanking sequences used in primer design in Table S4. To assess the degree to which Y-SNPs could discriminate western from other North American lineages (i.e., eastern, northwestern) and identify the western allele, we extended our Y analysis to include 361 reference samples collected from the eastern U.S. (*n* = 198), eastern Canada (*n* = 44), Alaska and northern Canada (*n* = 17), and five globally distributed fur farms (*n* = 102; Table S2). For each distinct Y-microsatellite haplotype (including reference samples), we selected 1–2 representative tissue samples to SNP-type (*n* = 128) and imputed SNP alleles for all other samples bearing an identical Y-microsatellite haplotype. We screened 21 candidate SNPs, four of which were previously shown to segregate within North America (Sacks et al. 2021); however, only one SNP was polymorphic in our dataset outside of Alaska (SNP-39) and was therefore the only one retained for final analysis. Upon identifying the putative native western allele for this Y-SNP, we then used a Fisher Exact test to compare the proportions of patrilines and matrilines in the Intermountain West that were western.

## Results

We obtained autosomal genotypes for 642 distinct individuals across nine western states (Table S1). All but three of the 31 loci significantly deviated from Hardy–Weinberg equilibrium as expected due to structure (Table S5). We also obtained 673 concatenated mitochondrial sequences that corresponded to 25 distinct haplotypes, two of which had novel D-loop sequences (A-278, sampled 5× in Utah, Genbank accession number OM810161; A-280, sampled 1× in Idaho, Genbank accession number OM810162) (Table S1). For the Y chromosome, we successfully typed 11–14 Y-microsatellites for 282 males and imputed Y-SNPs for 281 males in the western U.S., which corresponded to 32 distinct Y-haplotypes (Table S1).

### Autosomal population structure

**Fig. 2:**
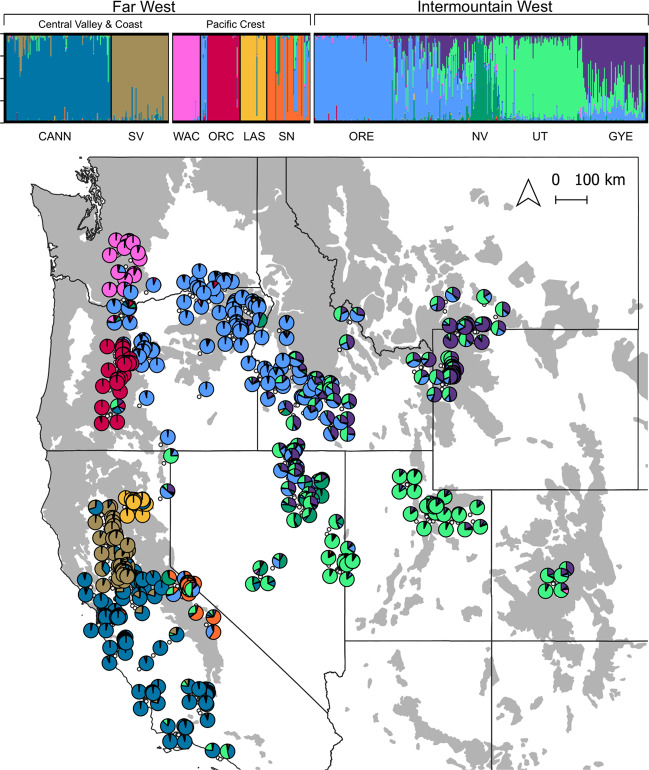
Population genetic structure of red foxes (*Vulpes vulpes*; *n* = 642) across the western contiguous U.S. based on 31 autosomal microsatellites, estimated by the spatially-explicit Bayesian clustering algorithm Tess at *K* = 10 genetic clusters. Admixture proportions for each individual are shown as bar plots (above) and spatially explicit pie charts (below). We categorized six clusters as discrete in the Far West and four clusters as continuous in the Intermountain West. Cluster abbreviations are CANN = California nonnative, GYE = Greater Yellowstone, LAS = Lassen Cascades, ORC = Oregon Cascades, ORE = eastern Oregon, NV = Nevada, UT = Utah, SN = Sierra Nevada, SV = Sacramento Valley, WAC = Washington Cascades.

All three clustering algorithms (Structure, Tess, DAPC) indicated broadly similar solutions, with 8–10 clusters encompassing the majority of structuring in genotype frequencies (Fig. S1). The *q* values assigned to individuals with respect to six of the clusters were stable across runs of *K* = 8, 9, or 10 clusters and, regardless of algorithm, were consistently >0.9 (Figs. S2–S6). The remaining ancestry was apportioned less consistently across runs to the other 2–4 clusters, with *q* values frequently falling between 0.25 and 0.75. To maximize resolution and because higher levels of *K* were consistently nested within lower levels of *K*, we based subsequent analyses on *K* = 10 (Fig. 2).

The six highly stable clusters with high *q* values corresponded to geographically discrete populations in the Far West: the Cascade subspecies in the Washington Cascades, each of the three populations of the Sierra Nevada subspecies (Oregon Cascades, Lassen, Sierra Nevada), the Sacramento Valley subspecies in California, and the known nonnative population of central and coastal California (Fig. 2). The four more variable clusters corresponded to red foxes primarily from the Intermountain West, with two exceptions: (1) several individuals from the Sierra Nevada DPS of Sierra Nevada red fox, and (2) all individuals from the Mount Hood (northernmost) region of the Oregon Cascades, both of which had *q* values >0.1 associated with the four Intermountain clusters. The former finding was expected based on previous studies that documented immigration from the Great Basin (Quinn et al. 2019). Otherwise, individuals in the Intermountain West were composed of mixed ancestry for which contribution varied continuously across geography: one cluster extended from eastern Oregon into western Idaho, another cluster primarily in the Greater Yellowstone area of Wyoming and Montana, a third cluster was most represented in Colorado and northeastern Utah, and a fourth cluster concentrated near the Ruby Mountains of northeastern Nevada (Fig. 2). In their totality, these patterns suggested discrete biological populations in the Far West and, by comparison, relatively continuous genetic structure across the Intermountain West.

### Genetic dissimilarity

**Fig. 3:**
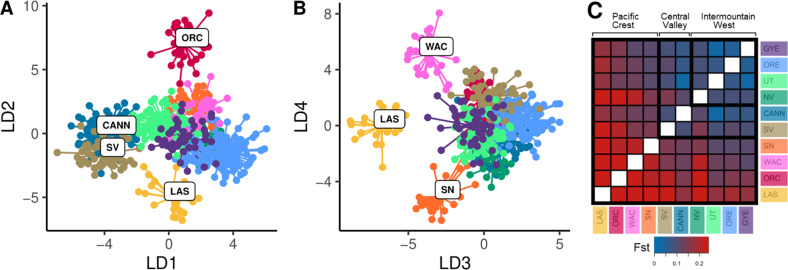
Genetic differentiation of red fox genetic clusters in the Western U.S. DAPC results according to (**A**) the first two linear discriminants (LD) and (**B**) the third and fourth LDs; (**C**) a matrix of pairwise *F*_ST_, with blues and reds indicating lower and higher *F*_ST_ values, respectively. In both analyses, abbreviations and colors correspond to cluster membership that was assigned using Tess admixture proportions in Fig. 2.

For clusters defined using Tess at *K* = 10, DAPC indicated higher levels of genetic dissimilarity for the six discrete clusters in the Far West relative to those in the Intermountain West (Fig. 3A, B). The first linear discriminant function separated the two low-elevation California populations (nonnative California, native Sacramento Valley) from all others (Fig. 3A), whereas the second through fourth discriminant functions isolated the four montane populations that reside in the Pacific mountains (Fig. 3B). Similarly, estimates of FST showed Pacific mountain clusters to be the most differentiated in all pairwise comparisons, especially in the Cascades (WAC = 0.12–0.25; ORC = 0.14–0.31; LAS = 0.16–0.31; SN = 0.10–0.24; Fig. 3C, Table S6). In contrast, clusters of the Intermountain West were completely overlapping in the first four linear functions of the DAPC and pairwise FST values among them were consistently low (0.04–0.10; Fig. 3A–C).

**Fig. 4:**
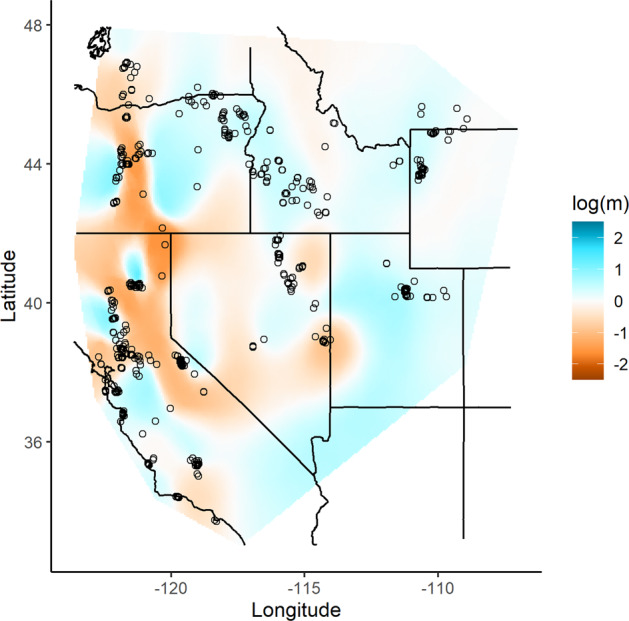
Effective migration surface is based on 637 samples (i.e., excluding five samples from Colorado) typed at 31 autosomal microsatellites. Effective migration surface for red foxes (Vulpes vulpes) in the western contiguous U.S. estimated using EEMS. Cool colors indicate areas with higher migration rates than expected under isolation-by-distance, warm colors indicate lower migration rates than expected, and white areas represent expectations under isolation-by-distance.

Results from EEMS corroborated the stronger degree of spatial structuring in the Far West, with substantially lower effective migration rates and particularly strong bands of resistance surrounding the four high-elevation populations in the Pacific mountains (Fig. 4). In the Intermountain West, the EEMS analysis indicated no significant resistance to gene flow between the higher elevation native historical range and more recent lower elevation expansion zones in the Intermountain West, suggesting genetic continuity.

### Geographic patterns of genetic diversity

**Fig. 5:**
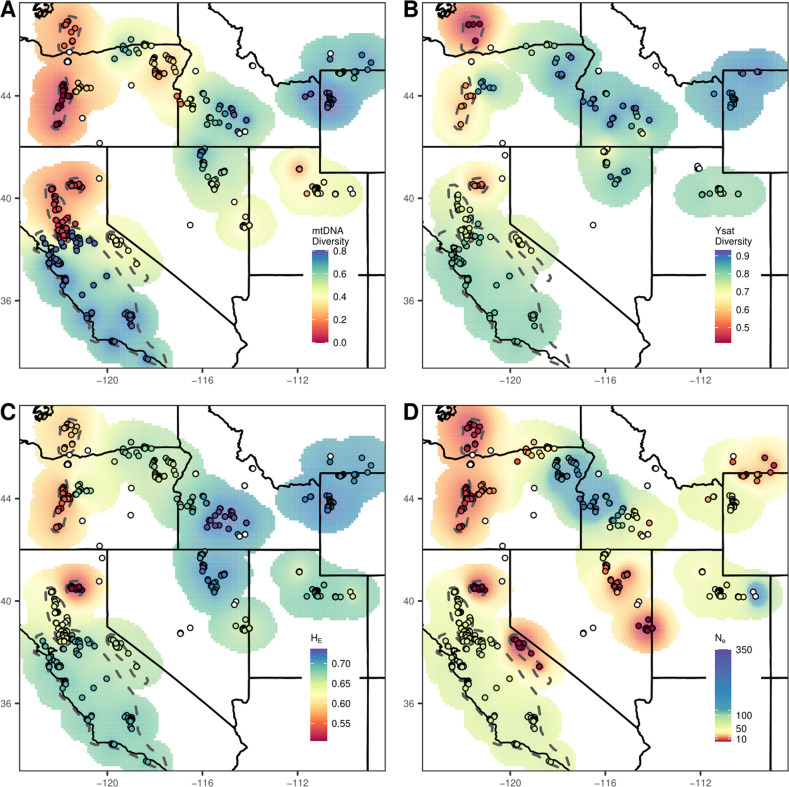
Spatiallly interpolated metrics of genetic diversity of red foxes (*Vulpes vulpes*) in the western contiguous U.S. **A** mitochondrial (mtDNA) gene diversity based on cytochrome *b* (including VVMC amplicon) and D-loop haplotypes (*n* = 626); (**B**) Y-chromosome gene diversity based on microsatellite haplotypes (*n* = 282); (**C**) expected heterozygosity (*H*_E_) for 31 autosomal microsatellites (*n* = 642); (**D**) genetic effective population sizes (*N*_e_) estimated using the bias-corrected linkage disequilibrium estimator with the same autosomal microsatellites. Diversity metrics were calculated for populations categorized as discrete according to spatial delineation of genetic clusters (dashed lines) and for all other populations using an overlapping neighborhood approach. White circles indicate neighborhoods with <10 samples (<5 for Y-chromosome diversity), for which estimations were not attempted.

Genetic diversity followed a similar overarching spatial pattern across marker types, in which the lowest values occurred in the Cascade Range of the Pacific mountains (Figs. 5, S7). Mitochondrial diversity showed the sharpest spatial contrasts between low- and high-diversity populations, as expected given female philopatry of red foxes (Gosselink et al. 2010) and the smaller effective population size of mitochondrial relative to autosomal loci (Fig. 5A). In particular, the Lassen Cascade and Oregon Cascade populations were nearly fixed for a single mitochondrial haplotype. The Y-chromosome haplotypes showed a similar pattern to mitochondrial haplotypes, although with the scale shifted higher, presumably due to a faster mutation rate of Y-microsatellites (Wei et al. 2013); In addition, the Washington Cascade population was the most depauperate in Y-chromosome haplotype diversity (Fig. 5B). Expected heterozygosity for autosomal microsatellites, an approximation of the genome average, similarly showed the populations in the Cascades to be the most genetically impoverished: Lassen with the lowest values (*H*_E_ = 0.51 ± 0.03 SE), followed by the Oregon Cascades (*H*_E_ = 0.55 ± 0.03 SE), and the Washington Cascades (*H*_E_ = 0.58 ± 0.03 SE; Fig. 5C, Table S7). For comparison, autosomal heterozygosities in the California lowlands and the Intermountain West ranged between 0.63 and 0.73.

Commensurate with genetic diversity, estimates of *N*_e_ were strikingly low in the Pacific mountains with point estimates of <10 for all four populations (Fig. 5D, Table S7). In contrast, estimates of *N*_e_ in the Intermountain West were higher and spatially heterogenous ($\underline x$ = 65, range = 3–352). Subsampling analyses indicated estimates within the Pacific mountains to be consistently small across subsampling trials, whereas estimates within the Intermountain West were highly sensitive to sampling scheme (Fig. S8). This suggests the heterogeneity of *N*_e_ estimates within the Intermountain West reflects a combination of true spatial variance and biases induced by gene flow and sample size (Waples and England 2011; Neel et al. 2013).

### Lineage introgression

**Fig. 6:**
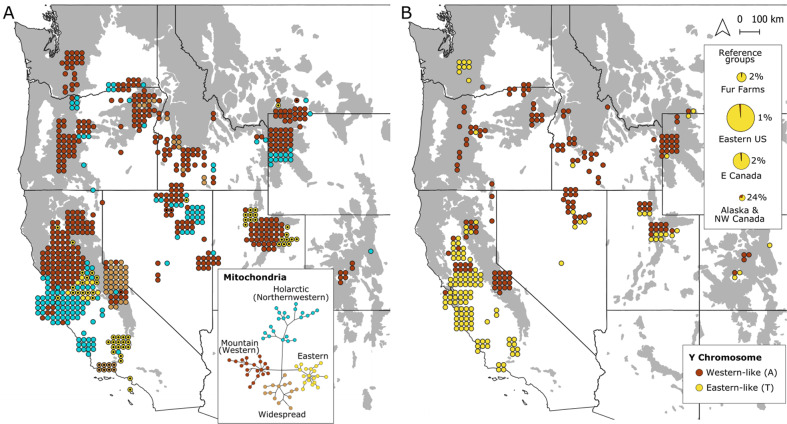
Lineage introgression of red foxes (*Vulpes vulpes*) in the western contiguous U.S. **A** mitochondrial matrilines (*n* = 673), with colors indicating phylogeographic clade and dots indicating matrilines sampled from fur farms. Inset shows median joining network used to determine phylogeographic clades. Matrilines belonging to the Mountain and Widespread subclades are assumed indigenous unless also sampled in fur farms; (**B**) Y-chromosome patrilines (*n* = 281), as informed by a single ancestry-informative SNP. Inset shows the frequency of the western-like allele in reference samples (*n* = 361) from globally distributed fur farms and wild North American populations.

Across the study area, most individuals possessed native mitochondrial matrilines belonging to either the Mountain or Widespread subclades (Fig. 6A, Table S8). Non-western matrilines were especially rare (3%) in Pacific mountain populations, the only exceptions being five individuals with a Holarctic matriline (G-38) in the northern Oregon Cascades that did not assign with more southern Cascade red foxes based on microsatellites, and two presumed fur-farm matrilines (G-38 and O-24) in the Sierra Nevada. Most samples in the Intermountain West also possessed native western matrilines, but nonnative haplotypes were comparatively more frequent (23%) there than in the Pacific mountains.

The Y-chromosome SNP-39 was nearly fixed (99%) for the T allele in eastern and fur-farm reference samples and of high frequency (76%) in the northern Alaska and northern Canada populations (Fig. 6B, Table S9). The nonnative population in California, known to be sourced from the eastern and northwestern lineages (Sacks et al. 2016), was also nearly fixed (99%) for the T allele. In contrast, most (72%) samples from the western U.S., excluding the nonnative California population, carried the A allele, suggesting SNP-39 acts as a reasonable discriminator of western ancestry. Under this assumption, the spatial patterning and frequency of introgressed patrilines were similar to those of the mitochondrial haplotypes (Fig. 6, Table S9), with the notable exceptions of the Washington Cascades and Sacramento Valley, where most males carried the “eastern-like” T allele despite the nearly exclusive occurrence of western matrilines in both regions. In the Intermountain West, non-western matrilines and patrilines occurred in the same localities and there was no significant difference in their frequencies (mtDNA = 23%, Y = 18%, *p* = 0.31). Importantly, only eight males in the western U.S. (outside the range of nonnative red foxes in California) carried both a non-western matriline and patriline (Table S1), supporting genetic admixture of indigenous and nonindigenous lineages in localities where non-western markers were clustered as opposed to pure nonindigenous red foxes.

### Fine-scaled structure in Oregon

**Fig. 7:**
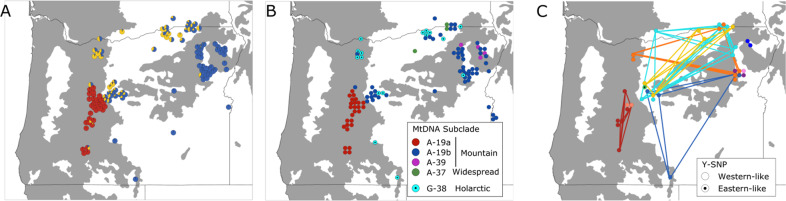
Genetic structuring of red foxes (*Vulpes vulpes*) in Oregon and southern Washington. Genetic structure according to (**A**) clustering of 120 autosomal microsatellite genotypes at *K* = 3 according to the spatially-explicit Bayesian clustering algorithm Tess; (**B**) 110 mitochondrial haplotypes with matrilineal clade indicated, and (**C**) 46 Y-microsatellite haplotypes. Shared Y-microsatellite haplotypes are connected by lines of the same color. Mitochondrial and Y-microsatellite haplotypes that are not native to the western U.S. are indicated with black dots.

Although we observed high connectivity across the broad Intermountain region, we sought to assess the possibility that the historical portion of the Rocky Mountain red fox range in Oregon retained its historical integrity, despite contributing gene flow to the lower-elevation expansion zone. In support of this scenario, analysis of fine-scaled autosomal variation suggested a distinction in the genetic composition of the lower and upper elevation populations that was not evident in the broader analysis. In contrast to the semi-continental-scale analysis that split Oregon into two genetically differentiated populations (southern Cascades versus all other locations), the fine-scale analysis of Oregon indicated greatest support for three genetic clusters (Fig. S9), corresponding to three geographic groups: (1) a cluster strongly associated with and restricted to the southern Cascades population, as in the broader analysis, (2) a cluster with high assignment values centered in the Wallowa and Blue Mountain ranges that correspond to the historical Rocky Mountain subspecific range (Bailey 1936b), and (3) a cluster restricted to lower elevations and the Mount Hood region (Fig. 7A). The low-elevation individuals additionally had a portion of ancestry attributed to the cluster corresponding with the historical Rocky Mountain red fox native range, whereas those in the native range generally did not have *q* values corresponding to the low-elevation cluster, suggesting the connectivity was unidirectional from high to low elevation. The enhanced resolution of this regional Oregon analysis also helped to clarify the relationship of the Mount Hood individuals with Intermountain ancestry. These individuals clustered with those in the lower elevation portion of Oregon rather than those in the historical native range of the Rocky Mountains, suggesting that the connectivity is a recent consequence of expansion across the Intermountain West rather than a reflection of ancient connectivity between native Rocky Mountain and Sierra Nevada red foxes.

Analysis of mitochondrial DNA also supported unidirectional gene flow from the historical range of the Rocky Mountain subspecies to the low-elevation basins. The two eastern Oregon clusters shared a native montane haplotype, but nearly all non-western haplotypes were relegated to individuals belonging to the low-elevation cluster (Fig. 7B). The Y chromosome similarly indicated gene flow between the historical and expansion zones in the form of shared Y-microsatellite haplotypes, but suggested differing compositions of patrilines (Fig. 7C). Red foxes in the native Rocky Mountain range contained three western patrilines not found at lower elevations, whereas the only patriline unique to the low-elevation expansion zone was indicated by its Y-SNP to be nonnative in origin.

Uniparentally inherited markers also supported autosomal analyses that showed red foxes in the Cascades as highly distinct from those found in the rest of the state. Most individuals in the core range shared matrilines and patrilines found only in the Cascades (Fig. 7B, C). As with autosomal DNA, the exception was near Mount Hood, where individuals shared matrilines and patrilines with red foxes at lower elevations in the expansion zone, consistent with recent gene flow.

## Discussion

The overarching impetus for our study was to inform the conservation of imperiled or potentially imperiled montane red fox populations of the Pacific mountains, which involved direct questions about those populations as well as about their relationship to and characteristics of adjacent populations in the Intermountain West. Broadly, the Pacific mountains contained isolated populations that were genetically distinct and depauperate of diversity, whereas red foxes in the Intermountain West showed low differentiation among regions and high genetic diversity, suggestive of higher gene flow, larger effective population sizes, or both. We found the transition from strong genetic structure in the Pacific mountains to the more weakly structured Intermountain West to be abrupt, with only low levels of genetic exchange between the two regional populations despite increasing proximity of expanding red fox populations in the low-elevation basins.

In contrast to the genetic discontinuities observed between Pacific and Intermountain regional populations of other montane species, which typically reflect isolation in different glacial refugia (Barrowclough et al. 2004; Manthey et al. 2012; Hope et al. 2016; Arbogast et al. 2017), the divisions observed in the present study were comparatively shallow and recent (e.g., <20,000 years; Aubry et al. 2009). Thus, our data suggest a contemporary genetic structure of western red foxes caused in large part by distinct anthropogenic factors. Native Pacific mountain populations likely became isolated as part of a general population decline precipitated by overharvest in the 19th and early 20th centuries (Perrine et al. 2010), whereas native montane populations in the Intermountain region likely colonized the cold desert basins and received admixture from translocated nonnative foxes during the mid-to late 1900s. Below, we discuss our findings and interpretations in the context of each of these regions separately.

### Genetic bottlenecks in the Pacific mountains

Our first objective was to identify whether red foxes in the southern Cascades (i.e., Oregon Cascade and Lassen populations) had low genetic diversity and effective population sizes, similarly to those previously documented in the Sierra Nevada and the northern Cascades of Washington (Akins et al. 2018; Quinn et al. 2019). All populations in the Pacific mountains, including Lassen and Oregon, had strikingly small genetic effective population sizes (*N*_e_ < 10). Microsatellite heterozygosities were also much lower (*H*_E_ = 0.51–0.58) in the Pacific mountains than other western regions. The exception was the Sierra Nevada DPS (*H*_E_ = 0.66), for which heterozygosity was only recently elevated by admixture with low-elevation foxes from the Great Basin (Quinn et al. 2019). Prior to immigration of foxes from the Intermountain region beginning in 2013, its heterozygosity estimated using the same marker set was the lowest of all western populations (*H*_E_ = 0.43, Quinn et al. 2019). As a benchmark for historical diversities, heterozygosities estimated from museum samples collected during 1850–1950 (based on a subset of 12 microsatellites) were 0.70 for the southern Cascades and 0.65 for the Sierra Nevada (Sacks et al. 2010). Together, the small effective population size and reduced heterozygosities imply a shared history of recent and dramatic bottlenecks, consistent with the hypothesis that unregulated harvest and poisoning associated with predator eradication activities during the 19th and 20th centuries were major contributors to population declines of montane red foxes (Perrine et al. 2010).

Evidence for severe genetic bottlenecks in all Pacific mountain populations has particular relevance for conservation of the Sierra Nevada subspecies, for which federal protection is currently restricted to the Sierra Nevada DPS (U.S. Fish and Wildlife Service 2021). At the time of initial review, no nuclear data were available from the Oregon Cascades and listing of the Southern Cascade DPS was declined based solely on a distribution of sighting records spanning the length of the Oregon Cascades (U.S. Fish and Wildlife Service 2015). Our findings raise a question as to what degree low genetic effective population sizes also reflect current abundance (i.e., census population size). At least two scenarios are possible with respect to contemporary populations in the Pacific mountains: (1) they are small and possibly decreasing in abundance, or (2) they are larger than suggested by their genetic effective population sizes and possibly increasing in abundance. In California, camera-based and noninvasive genetic scat surveys clearly indicate that Lassen and Sierra Nevada populations are composed of few individuals, relegated to a fraction of their historical range (SCAT, 2022). In Washington and Oregon, contemporary census population sizes are less certain, both in terms of abundance and trajectory (U.S. Fish and Wildlife Service 2015; Washington Department of Fish and Wildlife 2015). In the absence of abundance data, the low genetic effective population sizes and diversities observed in this study signal that the conservation status may not be all that more robust for the Cascade subspecies in Washington or the Southern Cascade DPS of the Sierra Nevada subspecies than it is for the endangered Sierra Nevada DPS.

In the meantime, our findings raise the possibility that regardless of poorly understood contemporary stressors (Perrine et al. 2010; SCAT, 2022), the genetic legacy of past declines may itself be contributing to a sluggish recovery in some or all Pacific mountain populations. Harvest is not considered to be a primary stressor because no harvest occurs in California and harvest is regulated in Oregon (SCAT, 2022). Furthermore, inbreeding depression has previously been demonstrated in the Sierra Nevada DPS (Quinn et al. 2019), raising the possibility that other Pacific populations with only slightly higher *N*_e_ may also experience reduced fitness, particularly relative to more outbred neighbors. Understanding the risks of inbreeding depression in other Pacific populations should be a research priority. Future study could involve pairing more precise estimates of inbreeding (e.g., using runs of homozygosity; Kardos et al. 2016) with variation in fitness-related traits among western populations

However, even without additional study, the current data are sufficient to implicate small population effects as a credible and immediate threat to the persistence of the Lassen population (e.g., following guidelines in Frankham et al. 2017). The genetic data definitively show that the Lassen and Oregon Cascade populations are not connected by long-distance dispersal, as suggested in previous assessments (U.S. Fish and Wildlife Service 2015), supporting near-total isolation of the Lassen population from other Pacific mountain populations. Combined with already diminished genetic diversity, as well as observations of interbreeding among first-order relatives and few documented individuals (SCAT, 2022), a strong case can be made for the likely benefits of genetic rescue to the Lassen population. In terms of safeguarding the “3Rs” (resiliency, representation, redundancy; Shaffer and Stein 2000), a foundational principle of the U.S. Endangered Species Act, extirpation of the Lassen population would compromise the redundancy and representation of the Sierra Nevada subspecies and Pacific mountain populations as a whole.

### Origin of red foxes in intermountain basins

Our second objective was to determine whether the recent appearance of red foxes in the cold desert basins of the Intermountain West could be explained by colonization of non-western lineages, as might be expected from habitat associations that differ from both historical and Pacific-mountain counterparts. We found that while inter-lineage admixture was present and at higher rates than in the Pacific mountains, the genomic composition of red foxes in the desert basins was predominantly native in origin. Frequencies of native western matrilines and patrilines were both high and roughly equivalent throughout low-elevation basins. In addition, we observed only weak nuclear differentiation between foxes in the historical range of the Rocky Mountain subspecies and those in expansion zones. Neither Bayesian clustering nor EEMS analyses differentiated these zones when the entire dataset was analyzed, implying either a high level of contemporary connectivity or a common founding source. These findings are consistent with conclusions of previous studies (e.g., Statham et al. 2012; Volkmann et al. 2015), but are based on a more comprehensive geographic and genomic dataset. Such downslope expansion of populations from the Rocky Mountains may have been facilitated by agricultural (e.g., irrigation) and other habitat conversion practices affecting prey abundance at lower elevations of the Intermountain region (Fichter and Williams 1967; Green et al. 2017). In addition, or alternatively, admixture itself (i.e., the exchange of particular genes) could have contributed to subsequent expansions of Rocky Mountain red foxes throughout lower elevation habitats.

Regardless of the driver, downslope expansion of populations likely increased proximity and opportunity for contact between native and nonnative red foxes in the Intermountain basins. Fine-scaled genetic analyses suggested low-level, but pervasive inter-lineage admixture in the expansion zone. When we repeated Bayesian clustering analyses at a local scale within Oregon (the region where higher sampling density allowed for more direct comparison of low- and high-elevation regions) structuring between historical and expansion zones was evident in both mitochondrial and nuclear genomes. These findings suggest the possibility that admixture was largely restricted to lower elevations, leaving genomes from higher elevation, montane regions in relatively “pure” native form. A negative correlation between elevation and admixture has been documented at a local scale in Colorado (Merson et al. 2017), but has not yet been tested throughout the Intermountain West. Increased sampling of more high-elevation montane regions in the Rocky Mountains and higher-powered genomic datasets (e.g., reduced representation-sequencing) could help elucidate more precisely how landscape features correspond to nonnative admixture.

The compositions of nonnative matrilines and patrilines were insufficient on their own to discriminate between fur-farm and wild midcontinent populations as the principal source of admixture. Both fur-farm stock and wild populations nearest the Rocky Mountains (e.g., Great Plains, Canadian taiga) are themselves expected to be composed of an admixture of northern and eastern lineages (Aubry et al. 2009; Statham et al. 2012; Black et al. 2018). Thus, while we sampled some matrilines common in fur farms (e.g., F-9 in the vicinity of Salt Lake City, UT; Fig. 6A), others (e.g., G-38; Table S8) were sampled in both translocated and wild midcontinent populations and were thus ambiguous in origin (Black et al. 2018). Tentatively, the spatial distribution of nonnative haplotypes supported fur-farm admixture over natural expansions. Nonnative haplotypes seemed more clustered than clinal, which is more congruent with localized pockets of feral fur-farm foxes near developed areas, as observed in lowland regions of California (Sacks et al. 2016) and Colorado (Merson et al. 2017), than an advancing directional wave (Kamler and Ballard 2002). The two hypotheses are not mutually exclusive; both natural and anthropogenic hybridization could be contributing to higher rates of inter-lineage admixture in the cold desert basins relative to the historical range.

### Conservation implications

Red foxes of the Intermountain West seem to be thriving and therefore are not of direct conservation concern. However, the potential effects of expanding low-elevation populations on Pacific mountain populations, both positive and negative, bear consideration. On one hand, low-to-moderate gene flow might be expected to alleviate inbreeding depression and boost short-term fitness levels (Frankham 2015; Whiteley et al. 2015), as observed in the first few generations following gene flow from the Great Basin into the Sierra Nevada population (Quinn et al. 2019). Conversely, the extreme disparity in population sizes makes the smaller Pacific mountain populations susceptible to swamping and disruption of local adaptations (Rhymer and Simberloff 1996; Harris et al. 2019; though see Fitzpatrick et al. 2020). A critical knowledge gap is how introgression from non-western lineages has altered the phenotype of admixed red foxes in the low-elevation Intermountain basins. Studies in the Greater Yellowstone Ecosystem found red foxes there to be considerably larger in body size than their Pacific mountain counterparts, suggesting a departure from the historical morphotype of montane red foxes (Roest 1977; Fuhrmann 1998; SCAT, 2022). Inter-lineage admixture could have introduced variation in other traits relevant to fitness in high-elevation environments as well (e.g., breeding phenology; Cross et al. 2018).

Given admixture in the Intermountain basins and its unknown effects on phenotype, we recommend that connectivity not be actively facilitated between Pacific mountain and low-elevation Intermountain regions. However, the expanding trajectory of red foxes in the Intermountain West suggests that contact with small populations in the Pacific mountains may be inevitable. As of yet, most Pacific mountain populations have retained their genetic distinctiveness despite the increasing proximity of low-elevation populations to their east. The isolation is especially notable in the southern portion of the Cascade Mountains in Oregon, where the native population showed little introgression from adjacent admixed basin red foxes well-within dispersal distance. In contrast, the federally endangered Sierra Nevada DPS showed more extensive gene flow with low-elevation Intermountain red foxes. Continued monitoring of contact zones will be necessary to determine whether differentiation is actively maintained by biological processes (e.g., Sacks et al. 2011) or represents a snapshot in time during the process of genetic homogenization.

More broadly, our findings highlight new complexities that can affect decisions about managing the genetic health and composition of endangered populations. A central paradigm in conservation genetics relates to balancing the risks of inbreeding and outbreeding depression in planned genetic rescue (Love Stowell et al. 2017; Ralls et al. 2018). Conversations about how to select donor individuals that will minimize outbreeding depression (e.g., swamping of local adaptations, increasing genetic load) while maximizing heterozygosity typically presuppose that managers have complete control of whether to conduct translocations, which populations to use as sources, and the rate at which to introduce individuals (e.g., Frankham et al. 2011, 2017; Kyriazis et al. 2021; Ralls et al. 2020). For managing potentially inbred Pacific mountain populations of red foxes, we face a more dynamic situation whereby an adjacent, increasing population currently or may soon contribute gene flow, regardless of its genetic suitability. The decision to intervene and actively translocate individuals from donor populations into Pacific mountain populations must weigh the usual risks, benefits, and costs, but also do so explicitly in the context of unmanaged gene flow from admixed Intermountain red foxes. Decision analyses will need to take into consideration rates of gene flow relative to effective population sizes, the importance of adaptive differences between Pacific mountain and low-elevation populations, and the capacity for selection to counter infusion of maladaptive alleles or allele combinations. Such assessments will ultimately inform the genetic management of Pacific mountain populations, requiring innovative modifications to a traditional conservation dilemma.

## Supplementary information

Supplemental Text and Figures

Supplemental Tables

## Data Availability

We have deposited the primary data underlying these analyses as follows: Autosomal microsatellite genotypes, Y-microsatellite genotypes, Y-SNP genotypes: Dryad (10.25338/B87P8K). Novel mitochondrial sequences: GenBank (OM810161–OM810162). Locations, Y-SNP identifiers, Y-microsatellite genotype identifiers, mitochondrial haplotype identifiers for samples in study area and reference samples: Supplementary Material Tables S1, S2.

## References

[CR1] Akins JR, Aubry KB, Sacks BN (2018). Genetic integrity, diversity, and population structure of the Cascade red fox. Conserv Genet.

[CR2] Allen SH, Sargeant AB (1993) Dispersal patterns of red foxes relative to population density. J Wildlife Manag 57(3):526–533

[CR3] Arbogast BS, Schumacher KI, Kerhoulas NJ, Bidlack AL, Cook JA, Kenagy GJ (2017). Genetic data reveal a cryptic species of New World flying squirrel: Glaucomys oregonensis. J Mammal.

[CR4] Ashbrook FG (1928) Silver-Fox Farming. US Department of Agriculture. Washington D.C.

[CR5] Aubry KB (1984). The recent history and present distribution of the red fox in Washington. Northwest Sci.

[CR6] Aubry KB, Statham MJ, Sacks BN, Perrine JD, Wisely SM (2009). Phylogeography of the North American red fox: vicariance in Pleistocene forest refugia. Mol Ecol.

[CR7] Bailey V (1936). The red fox in America. Nature.

[CR8] Bailey V (1936). The Mammals and Life Zones of Oregon.

[CR9] Ball MC, Finnegan L, Manseau M, Wilson P (2010). Integrating multiple analytical approaches to spatially delineate and characterize genetic population structure: an application to boreal caribou (*Rangifer tarandus caribou*) in central Canada. Conserv Genet.

[CR10] Barrowclough GF, Groth JG, Mertz LA, Gutiérrez RJ (2004). Phylogeographic structure, gene flow and species status in blue grouse (*Dendragapus obscurus*). Mol Ecol.

[CR11] Bell DA, Robinson ZL, Funk WC, Fitzpatrick SW, Allendorf FW, Tallmon DA (2019). The exciting potential and remaining uncertainties of genetic rescue. Trends Ecol Evol.

[CR12] Black KL, Petty SK, Radeloff VC, Pauli JN (2018). The Great Lakes Region is a melting pot for vicariant red fox (*Vulpes vulpes*) populations. J Mammal.

[CR13] Champagnon J, Elmberg J, Guillemain M, Gauthier-Clerc M, Lebreton JD (2012). Conspecifics can be aliens too: a review of effects of restocking practices in vertebrates. J Nat Conserv.

[CR14] Chen C, Durand E, Forbes F, François O (2007). Bayesian clustering algorithms ascertaining spatial population structure: a new computer program and a comparison study. Mol Ecol Notes.

[CR15] Cross PR, Sacks BN, Luikart G, Schwartz MK, Van Etten KW, Crabtree RL (2018). Red Fox Ancestry and Connectivity Assessments Reveal Minimal Fur Farm Introgression in Greater Yellowstone Ecosystem. J Fish Wildl Manag.

[CR16] Dalquest, WW (1948) Mammals of Washington, Vol II. University of Kansas Publications, Museum of Natural History, Lawrence, Kansas

[CR17] Devenish-Nelson ES, Harris S, Soulsbury CD, Richards SA, Stephens PA (2013). Demography of a carnivore, the red fox, *Vulpes vulpes*: what have we learnt from 70 years of published studies?. Oikos.

[CR18] Do C, Waples RS, Peel D, Macbeth G, Tillett BJ, Ovenden JR (2014). NeEstimator v2: re-implementation of software for the estimation of contemporary effective population size (Ne) from genetic data. Mol Ecol Resour.

[CR19] Durand E, Jay F, Gaggiotti OE, François O (2009). Spatial inference of admixture proportions and secondary contact zones. Mol Biol Evol.

[CR20] Earl DA, vonHoldt BM (2012). Structure Harvester: a website and program for visualizing Structure output and implementing the Evanno method. Conserv Genet Resour.

[CR21] Edmands S (2007). Between a rock and a hard place: evaluating the relative risks of inbreeding and outbreeding for conservation and management. Mol Ecol.

[CR22] Falush D, Stephens M, Pritchard JK (2003). Inference of population structure using multilocus genotype data: linked loci and correlated allele frequencies. Genetics.

[CR23] Fichter E, Williams R (1967). Distribution and status of the red fox in Idaho. J Mammal.

[CR24] Fitzpatrick SW, Bradburd GS, Kremer CT, Salerno PE, Angeloni LM, Funk WC (2020). Genomic and fitness consequences of genetic rescue in wild populations. Curr Biol.

[CR25] Frankham R (1996). Relationship of genetic variation to population size in wildlife. Conserv Biol.

[CR26] Frankham R (2015). Genetic rescue of small inbred populations: Meta-analysis reveals large and consistent benefits of gene flow. Mol Ecol.

[CR27] Frankham R, Ballou JD, Eldridge MD, Lacy RC, Ralls K, Dudash MR, Fenster CB (2011). Predicting the probability of outbreeding depression. Conserv Biol.

[CR28] Frankham R, Ballou JD, Ralls K, Eldridge MD, Dudash MR, Fenster CB (2017). Genetic management of fragmented animal and plant populations.

[CR29] Fuhrmann RT (1998) Distribution, Morphology, and Habitat Use of the Red Fox in the Northern Yellowstone Ecosystem. MSc Thesis, Montana State University, Bozeman, Montana

[CR30] Gortázar C, Travaini A, Delibes M (2000). Habitat-related microgeographic body size variation in two Mediterranean populations of red fox (*Vulpes vulpes*). J Zool Lond.

[CR31] Gosselink TE, Piccolo KA, Van Deelen TR, Warner RE, Mankin PC (2010). Natal dispersal and philopatry of red foxes in urban and agricultural areas of Illinois. J Wildl Manag.

[CR32] Goudet J (2005). Hierfstat, a package for R to compute and test hierarchical *F*-statistics. Mol Ecol Notes.

[CR33] Green GA, Sacks BN, Erickson LJ, Aubry KB (2017). Genetic characteristics of red foxes in northeastern Oregon. Northwest Naturalist.

[CR34] Grinnell J, Dixon JS, Linsdale JM (1937). Fur-Bearing Mammals of California, Vol II.

[CR35] Hall E, Kelson KR (1959). The Mammals of North America. 2 Vols.

[CR36] Hampe A, Petit RJ (2005). Conserving biodiversity under climate change: the rear edge matters. Ecol Lett.

[CR37] Harris K, Zhang Y, Nielsen R (2019). Genetic rescue and the maintenance of native ancestry. Conserv Genet.

[CR38] Hedrick PW, Peterson RO, Vucetich LM, Adams JR, Vucetich JA (2014). Genetic rescue in Isle Royale wolves: genetic analysis and the collapse of the population. Conserv Genet.

[CR39] Hiller TL, McFadden-Hiller JE, Sacks BN (2015). Genetic and photographic detections document Sierra Nevada red fox in the Northern Cascades of Oregon. Northwest Sci.

[CR40] Hoffmann M, Sillero-Zubiri C (2021). Vulpes vulpes (amended version of 2016 assessment). IUCN Red List Threatened Species.

[CR41] Hoffmann RS, Wright PL, Newby FE (1969). The distribution of some mammals in Montana I. Mammals other than bats. J Mammal.

[CR42] Hope AG, Malaney JL, Bell KC, Salazar-Miralles F, Chavez AS, Barber BR (2016). Revision of widespread red squirrels (genus: *Tamiasciurus*) highlights the complexity of speciation within North American forests. Mol Phylogenet Evol.

[CR43] Jakobsson M, Rosenberg NA (2007). CLUMPP: a cluster matching and permutation program for dealing with label switching and multimodality in analysis of population structure. Bioinformatics.

[CR44] Jombart T (2008). adegenet: a R package for the multivariate analysis of genetic markers. Bioinformatics.

[CR45] Jombart T, Devillard S, Balloux F (2010). Discriminant analysis of principal components: a new method for the analysis of genetically structured populations. BMC Genet.

[CR46] Kamler JF, Ballard WB (2002) A review of native and nonnative red foxes in North America. Wildlife Soc Bullet 30(2):370–379

[CR47] Kamler JF, Ballard WB (2003) Range expansion of red foxes in eastern Nevada and western Utah. J Arizona-Nevada Acad Sci 36(1):18–20

[CR48] Kardos M, Taylor HR, Ellegren H, Luikart G, Allendorf FW (2016). Genomics advances the study of inbreeding depression in the wild. Evolut Appl.

[CR49] Kasprowicz AE, Statham MJ, Sacks BN (2016). Fate of the other redcoat: remnants of colonial British foxes in the eastern United States. J Mammal.

[CR50] Kuchler A (1964) Potential natural vegetation of the conterminous United States. Am Geogr Soc Spec Publ 36:1–116

[CR51] Kyriazis CC, Wayne RK, Lohmueller KE (2021). Strongly deleterious mutations are a primary determinant of extinction risk due to inbreeding depression. Evol Lett.

[CR52] Laikre L, Schwartz MK, Waples RS, Ryman N, Group GW (2010). Compromising genetic diversity in the wild: unmonitored large-scale release of plants and animals. Trends Ecol Evol.

[CR53] Larivière S, Pasitschniak-Arts M (1996). Vulpes vulpes. Mamm Species.

[CR54] Laut AC (1921). The fur trade of America.

[CR55] Leigh JW, Bryant D (2015). Popart: full-feature software for haplotype network construction. Methods Ecol Evol.

[CR56] Lesica P, Allendorf FW (1995). When are peripheral populations valuable for conservation?. Conserv Biol.

[CR57] Lewis JC, Sallee KL, Golightly Jr RT (1999) Introduction and range expansion of nonnative red foxes (*Vulpes vulpes*) in California. Am Midland Naturalist 142(2):372–381

[CR58] Long J (2003). Introduced mammals of the world: their history, distribution and influence.

[CR59] Lord KA, Larson G, Coppinger RP, Karlsson EK (2020). The history of farm foxes undermines the animal domestication syndrome. Trends Ecol Evol.

[CR60] Love Stowell SM, Pinzone CA, Martin AP (2017). Overcoming barriers to active interventions for genetic diversity. Biodivers Conserv.

[CR61] Lounsberry ZT, Quinn CB, Statham MJ, Angulo CL, Kalani TJ, Tiller E (2017). Investigating genetic introgression from farmed red foxes into the wild population in Newfoundland, Canada. Conserv Genet.

[CR62] Mace RU (1970). Oregon’s Furbearing Animals. Oregon State Game Commission, Corvallis, Oregon

[CR63] Manthey JD, Klicka J, Spellman GM (2012). Is gene flow promoting the reversal of Pleistocene divergence in the Mountain Chickadee (Poecile gambeli)?. PLOS ONE.

[CR65] Merson C, Statham MJ, Janecka JE, Lopez RR, Silvy NJ, Sacks BN (2017). Distribution of native and nonnative ancestry in red foxes along an elevational gradient in central Colorado. J Mammal.

[CR66] Miller JM, Cullingham CI, Peery RM (2020). The influence of a priori grouping on inference of genetic clusters: simulation study and literature review of the DAPC method. Heredity.

[CR67] Moore M, Brown SK, Sacks BN (2010). Thirty-one short red fox (*Vulpes vulpes*) microsatellite markers. Mol Ecol Resour.

[CR68] Moritz C (1994). Applications of mitochondrial DNA analysis in conservation: a critical review. Mol Ecol.

[CR69] Moritz C (2002). Strategies to protect biological diversity and the evolutionary processes that sustain it. Syst Biol.

[CR70] Neel MC, McKelvey K, Ryman N, Lloyd MW, Bull RS, Allendorf FW (2013). Estimation of effective population size in continuously distributed populations: there goes the neighborhood. Heredity.

[CR71] Nei M (1987). Molecular Evolutionary Genetics.

[CR72] Pebesma EJ (2004). Multivariable geostatistics in S: the gstat package. Comput Geosci.

[CR73] Paradis E (2010). pegas: an R package for population genetics with an integrated–modular approach. Bioinformatics.

[CR74] Perrine JD, Campbell LA, Green GA (2010) Sierra Nevada red fox (*Vulpes vulpes necator*): a conservation assessment. US Department of Agriculture, Vallejo, California

[CR75] Perrine JD, Pollinger JP, Sacks BN, Barrett RH, Wayne RK (2007). Genetic evidence for the persistence of the critically endangered Sierra Nevada red fox in California. Conserv Genet.

[CR76] Petersen M (1914). The fur traders and fur bearing animals.

[CR77] Petkova D, Novembre J, Stephens M (2016). Visualizing spatial population structure with estimated effective migration surfaces. Nat Genet.

[CR78] Petkova (2020) reemsplots2: Generate plots to inspect and visualize the results of EEMS. R package version 0.1.0. https://github.com/dipetkov/eems

[CR79] Pritchard JK, Stephens M, Donnelly P (2000). Inference of population structure using multilocus genotype data. Genetics.

[CR80] Pritchard JK, Wen W, Falush D (2003) Documentation for STRUCTURE Software: Version 2. https://web.stanford.edu/group/pritchardlab/software/readme_structure2.pdf Accessed 3 Dec 2020

[CR81] Quinn CB, Alden PB, Sacks BN (2019). Noninvasive sampling reveals short-term genetic rescue in an insular red fox population. J Heredity.

[CR82] Ralls K, Ballou JD, Dudash MR, Eldridge MD, Fenster CB, Lacy RC (2018). Call for a paradigm shift in the genetic management of fragmented populations. Conserv Lett.

[CR83] Ralls K, Sunnucks P, Lacy RC, Frankham R (2020). Genetic rescue: a critique of the evidence supports maximizing genetic diversity rather than minimizing the introduction of putatively harmful genetic variation. Biol Conserv.

[CR84] Rando HM, Stutchman JT, Bastounes ER, Johnson JL, Driscoll CA, Barr CS (2017). Y-chromosome Markers for the Red Fox. J Heredity.

[CR85] Rhymer JM, Simberloff D (1996). Extinction by hybridization and introgression. Annu Rev Ecol Syst.

[CR86] Roest AI (1977) Taxonomic status of the red fox in California. State of California, The Resources Agency, Department of Fish and Game, California Polytechnic State University, San Luis Obispo, California

[CR87] Roux C, Fraisse C, Romiguier J, Anciaux Y, Galtier N, Bierne N (2016). Shedding light on the grey zone of speciation along a continuum of genomic divergence. PLOS Biol.

[CR88] Sacks BN, Brazeal JL, Lewis JC (2016). Landscape genetics of the nonnative red fox of California. Ecol Evol.

[CR89] Sacks B, Lounsberry Z, Rando H, Kluepfel K, Fain S, Brown S (2021). Sequencing red fox Y chromosome fragments to develop phylogenetically informative SNP markers and glimpse male-specific trans-Pacific phylogeography. Genes.

[CR90] Sacks BN, Moore M, Statham MJ, Wittmer HU (2011). A restricted hybrid zone between native and introduced red fox (*Vulpes vulpes*) populations suggests reproductive barriers and competitive exclusion. Mol Ecol.

[CR91] Sacks BN, Statham MJ, Perrine JD, Wisely SM, Aubry KB (2010). North American montane red foxes: expansion, fragmentation, and the origin of the Sacramento Valley red fox. Conserv Genet.

[CR92] Saunders G, Coman B, Kinnear J, Braysher M (1995). Managing vertebrate pests: foxes.

[CR93] Sierra Nevada Red Fox Conservation Advisory Team [SCAT] (2022) A Conservation Strategy for the Sierra Nevada Red Fox. California Department of Fish and Wildlife, Sacramento, USA, In press

[CR94] Seton E (1929). Lives of Game Animals.

[CR95] Shaffer ML, Stein BA, Stein BA, Kutner LS, Adams JS (2000). Safeguarding our precious heritage. Precious heritage: the status of biodiversity in the United States.

[CR96] Shirk A, Cushman S (2011). sGD: software for estimating spatially explicit indices of genetic diversity. Mol Ecol Resour.

[CR97] Sikes RS, Gannon WL, Animal Care and Use Committee of the American Society of Mammalogists (2011). Guidelines of the American Society of Mammalogists for the use of wild mammals in research. J Mammal.

[CR98] Statham MJ, Murdoch J, Janecka J, Aubry KB, Edwards CJ, Soulsbury CD (2014). Range-wide multilocus phylogeography of the red fox reveals ancient continental divergence, minimal genomic exchange and distinct demographic histories. Mol Ecol.

[CR99] Statham MJ, Sacks BN, Aubry KB, Perrine JD, Wisely SM (2012). The origin of recently established red fox populations in the United States: translocations or natural range expansions?. J Mammal.

[CR100] Statham MJ, Trut LN, Sacks BN, Kharlamova AV, Oskina IN, Gulevich RG (2011). On the origin of a domesticated species: identifying the parent population of Russian silver foxes (Vulpes vulpes). Biol J Linn Soc.

[CR101] Szuma E (2008). Evolutionary and climatic factors affecting tooth size in the red fox *Vulpes vulpes* in the Holarctic. Mammal Res.

[CR102] U.S. Fish and Wildlife Service (2015). Endangered and threatened wildlife and plants; 12-month finding on a petition to list Sierra Nevada red fox as an endangered or threatened species. Fed Reg.

[CR103] U.S. Fish and Wildlife Service (2021). Endangered and threatened wildlife and plants; endangered status for the Sierra Nevada Distinct Population Segment of the Sierra Nevada red fox. Fed Reg.

[CR104] van der Valk T, de Manuel M, Marques-Bonet T, Guschanski K (2021) Estimates of genetic load suggest frequent purging of deleterious alleles in small populations. bioRxiv:696831

[CR105] Verts B, Carraway LN (1998). Land Mammals of Oregon.

[CR106] Volkmann LA, Statham MJ, Mooers AØ, Sacks BN (2015). Genetic distinctiveness of red foxes in the Intermountain West as revealed through expanded mitochondrial sequencing. J Mammal.

[CR107] Waples RS (2006). A bias correction for estimates of effective population size based on linkage disequilibrium at unlinked gene loci. Conserv Genet.

[CR108] Waples RS, Antao T, Luikart G (2014). Effects of overlapping generations on linkage disequilibrium estimates of effective population size. Genetics.

[CR109] Waples RS, Do C (2008). LDNE: a program for estimating effective population size from data on linkage disequilibrium. Mol Ecol Resour.

[CR110] Waples RS, Do C (2010). Linkage disequilibrium estimates of contemporary Ne using highly variable genetic markers: a largely untapped resource for applied conservation and evolution. Evolut Appl.

[CR111] Waples RS, England PR (2011). Estimating contemporary effective population size on the basis of linkage disequilibrium in the face of migration. Genetics.

[CR112] Washington Department of Fish and Wildlife (2015). Washington’s State Wildlife Action Plan: 2015 Update.

[CR113] Wei W, Ayub Q, Xue Y, Tyler-Smith C (2013). A comparison of Y-chromosomal lineage dating using either resequencing or Y-SNP plus Y-STR genotyping. Forensic Sci Int- Genet.

[CR114] Weir BS, Cockerham CC (1984) Estimating F-statistics for the analysis of population structure. Evolution 36:1358–1370 10.1111/j.1558-5646.1984.tb05657.x 28563791

[CR115] Whiteley AR, Fitzpatrick SW, Funk WC, Tallmon DA (2015). Genetic rescue to the rescue. Trends Ecol Evol.

[CR116] Wilder AP, Navarro AY, King SN, Miller WB, Thomas SM, Steiner CC (2020). Fitness costs associated with ancestry to isolated populations of an endangered species. Conserv Genet.

[CR117] Wright S (1946). Isolation by distance under diverse systems of mating. Genetics.

[CR118] Zabel CJ, Taggart SJ (1989). Shift in red fox, *Vulpes vulpes*, mating system associated with El Niño in the Bering Sea. Anim Behav.

