# Supplementary material for: Contrasting genetic trajectories of endangered and expanding red fox populations in the western U.S
Source: Heredity (Edinb). 2022 Mar 21;129(2):123–36. doi: 10.1038/s41437-022-00522-4 (PMC9338314; doi:10.1038/s41437-022-00522-4)
Supplement: Supplementary file 1 — Supplemental Text and Figures [file 41437_2022_522_MOESM1_ESM.pdf]

Quinn et al. Contrasting genetic trajectories of endangered and expanding red fox populations in the western U.S. Supplementary Material

Supplementary Text

Taxonomy of western red foxes

Merriam (1900) divided the native western lineage of red fox (*Vulpes vulpes*) into three geographically defined subspecies: the Sierra Nevada red fox (*V. v. necator*), the Cascade red fox (*V. v. cascadensis*), and the Rocky Mountain red fox (*V. v. macroura*; Fig. 1 in main text). Originally the range of the Cascade red fox included the Cascades of both Oregon and Washington (Bailey 1936; Hall and Kelson 1959), but more recent genetic analyses indicated that red foxes in the Oregon Cascades were more closely related to those in California than in Washington (Sacks et al. 2010b). Subsequently, the range of the Sierra Nevada red fox was expanded to include the Pacific mountains (i.e., Cascade Ranges) of Oregon as well as California (Cascade and Sierra Nevada ranges).

A fourth subspecies of the native western lineage was discovered in the semi-arid grasslands of California's Sacramento Valley (*V. v. patwin;* Grinnell et al. 1937; Sacks et al. 2010b*)* and is the only native western representative with a historically described low elevation distribution. Throughout most of the 20th century, red foxes of the Sacramento Valley were speculated to be nonnative red foxes that originated from anthropogenic translocations (Grinnell et al. 1937; Roest 1977; Lewis et al. 1999), but genetic analyses have since revealed endemic mitochondrial haplotypes and a sister relationship to the Sierra Nevada red fox (Sacks et al. 2010b). Sacramento Valley red foxes therefore also belong to the indigenous western lineage of red foxes, despite being ecologically and phenotypically distinct from the other, predominantly montane native subspecies (Sacks et al. 2010a).

Mitchondrial analyses suggest that all members of the western lineage were likely connected through gene flow at the time of the Last Glacial Maximum (Aubry et al. 2009). It is not known when subspecies became isolated into disjunct subspecific ranges, but this likely occurred during the Holocene.

Conservation status of western red fox populations

The Cascade subspecies of Washington is currently a candidate for state-listing in Washington due to its restricted distribution (Washington Department of Fish and Wildlife 2013). The Sierra Nevada subspecies was state-listed as a "Threatened" species in California in 1980 and has been protected from hunting and trapping since 1974 (California Department of Fish and Game 2005). In 2015, the U.S. Fish and Wildlife Service recognized the Sierra Nevada red fox as being composed of two distinct population segments (DPS): (1) the Sierra Nevada DPS in the Sierra Nevada range (2) the Southern Cascade DPS, composed of populations in the vicinity of Lassen Peak of northern California and the Oregon Cascades (U.S. Fish and Wildlife Service 2015). The USFWS designated the Sierra Nevada DPS as federally endangered (U.S. Fish and Wildlife Service 2021), but determined the Southern Cascade DPS as not warranted for listing, largely due to an absence of data in the Oregon Cascades at that time (U.S. Fish and

Wildlife Service 2015). The Sacramento Valley red fox is state-listed as a California Mammal Species of Greatest Conservation Need (California Department of Fish and Wildlife 2015). The Rocky Mountain red fox currently does not have any conservation status.

Literature cited

Aubry KB, Statham MJ, Sacks BN, Perrine JD, Wisely SM (2009) Phylogeography of the North American red fox: vicariance in Pleistocene forest refugia. Molecular Ecology 18(12): 2668-2686

Bailey V (1936) The Mammals and Life Zones of Oregon. U.S. Government Printing Office: Washington, D.C.

California Department of Fish and Game (2005) The status of rare, threatened, and endangered plants and animals of California 2000–2004. California Department of Fish and Game, Sacramento, USA.

California Department of Fish and Wildlife (2015) in A. G. Gonzales and J. Hoshi, editors. California State Wildlife Action Plan, 2015 update: a conservation legacy for Californians. Ascent Environmental, Inc., Sacramento, California, USA.

Grinnell J, Dixon JS, Linsdale JM (1937) Fur-Bearing Mammals of California, Vol II. University of California Press: Berkeley, California

Hall E, Kelson KR (1959) The Mammals of North America. 2 Vols. Ronald Press, New York

Lewis JC, Sallee KL, Golightly Jr RT (1999) Introduction and range expansion of nonnative red foxes (*Vulpes vulpes*) in California. American Midland Naturalist: 372-381

Roest AI (1977) Taxonomic Status of the Red Fox in California. State of California, The Resources Agency, Department of Fish and Game

Sacks BN, Wittmer HU, and Statham MJ (2010a) The Native Sacramento Valley red fox. Report to the California Department of Fish and Game, May 30th, 2010, 49 pp. http://mecu.faculty.ucdavis.edu/wp-content/uploads/sites/491/2017/11/The-Native-Sacramento-ValleyRedFox_2010.pdf [Accessed December 2021]

Sacks BN, Statham MJ, Perrine JD, Wisely SM, Aubry KB (2010b) North American montane red foxes: expansion, fragmentation, and the origin of the Sacramento Valley red fox. Conservation Genetics 11(4): 1523-1539

U.S. Fish and Wildlife Service (2015) Endangered and threatened wildlife and plants; 12-month finding on a petition to list Sierra Nevada red fox as an endangered or threatened species. Fed Reg 80:60989-61028

U.S. Fish and Wildlife Service (2021) Endangered and threatened wildlife and plants; endangered status for the Sierra Nevada Distinct Population Segment of the Sierra Nevada red fox. Fed Reg 86: 41743–41758

Washington Department of Fish and Wildlife (2013) Threatened and Endangered Wildlife in Washington: 2012 Annual Report. Listing and Recovery Section, Wildlife Program, Washington Department of Fish and Wildlife, Olympia, Washington, USA. 251 pp

Supplementary Figures

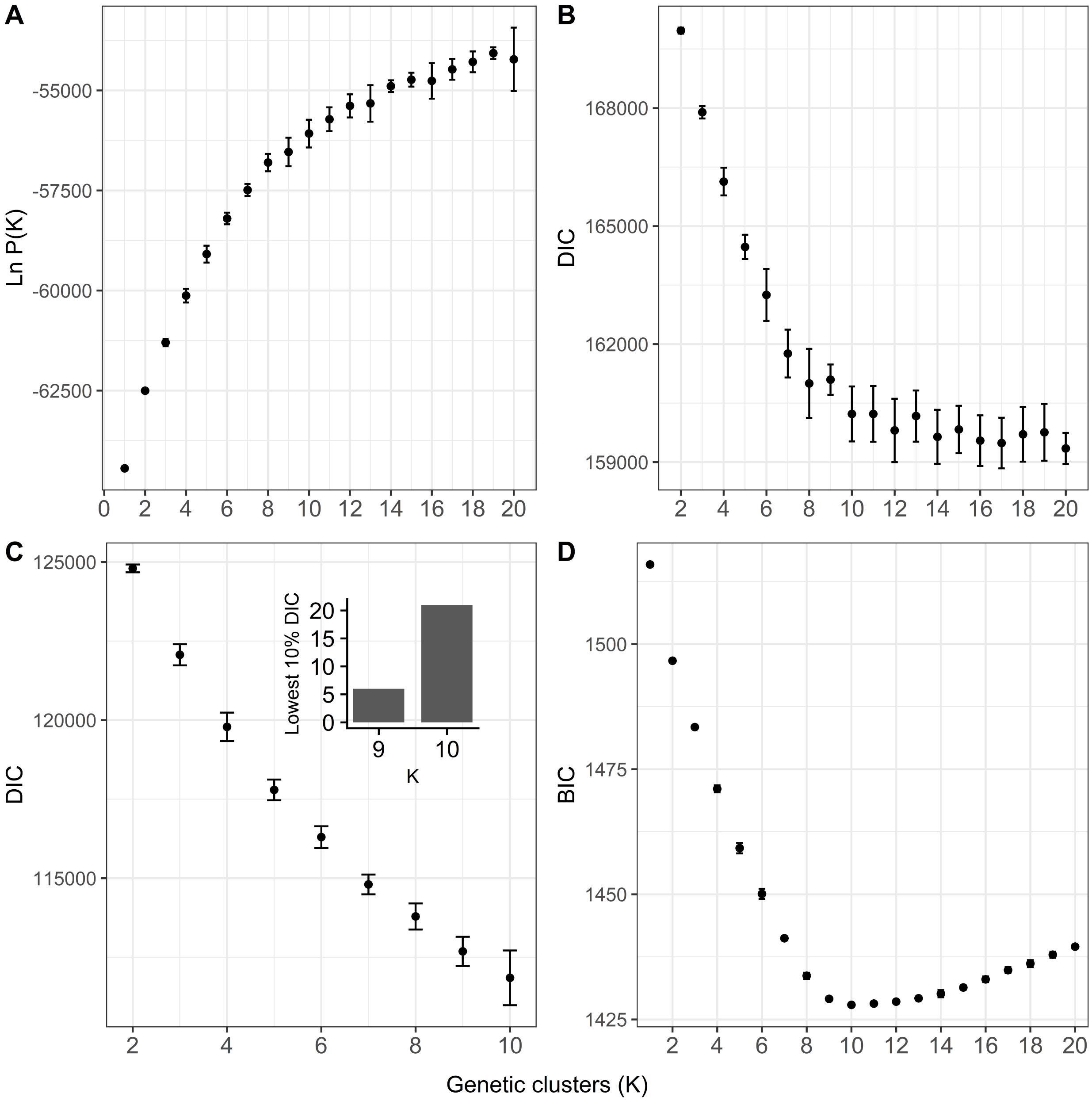

Figure S1. Performance of three different clustering algorithms used to evaluate the appropriate number of genetic clusters (K): (A) the mean log probability of the data for Structure, the deviance information criteria (DIC) for TESS runs with (B) no admixture and (C) admixture, and (D) the Bayesian information criteria (BIC) for DAPC analysis. Considered together, algorithms indicated the strongest support for 8–10 genetic clusters.

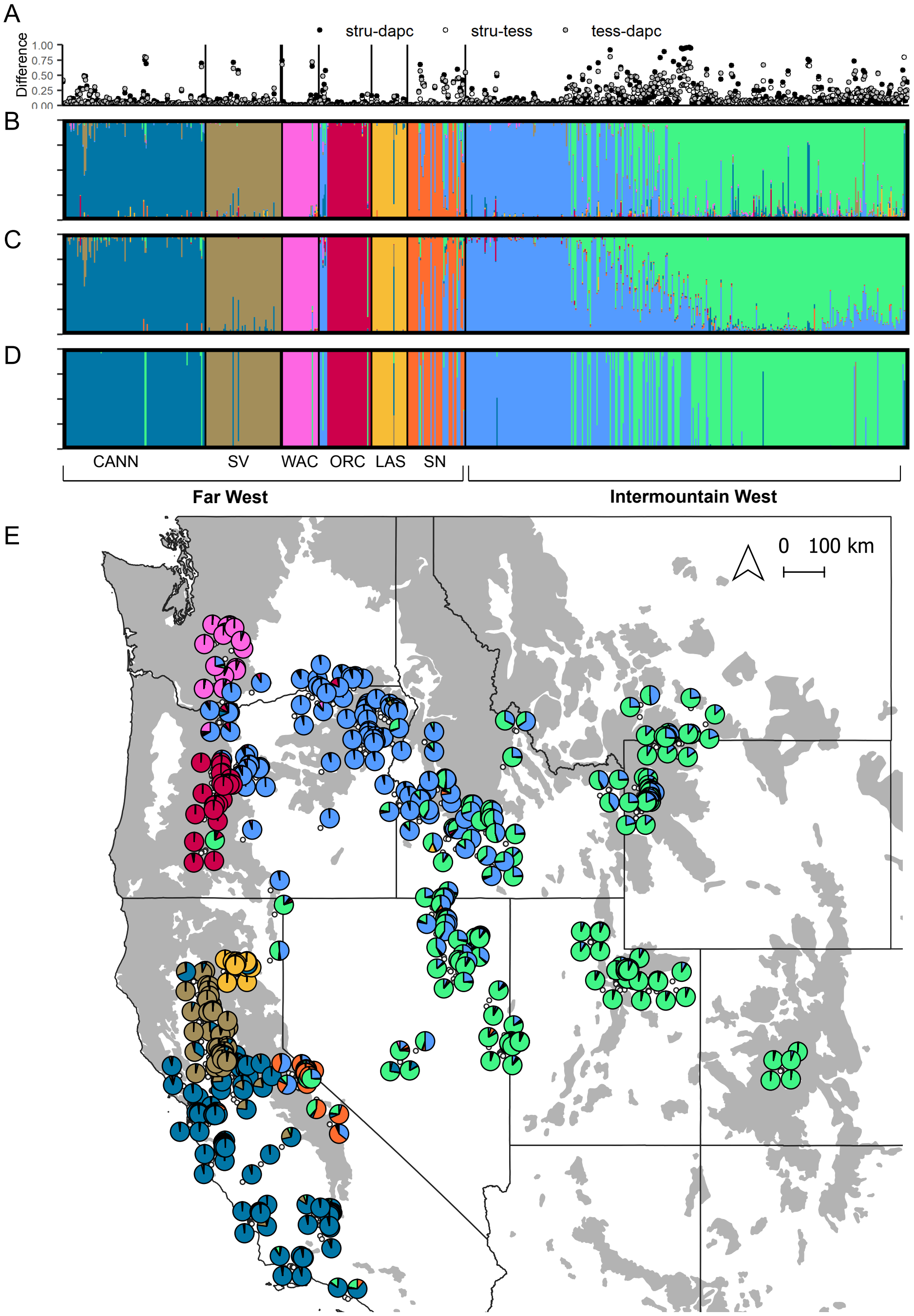

Figure S2. Genetic analysis of 642 red foxes in the western U.S., according to 31 autosomal microsatellites, at K = 8 genetic clusters. (A) Pairwise differences between individual ancestry proportions (q) determined by different algorithms, bar plots of clustering solutions from (B) Structure, (C) Tess, and (D) DAPC, and (E) pie charts showing the geographic distribution of TESS clusters. Solutions are broadly similar among methods, but there are more discrepancies in q values for individuals from the Intermountain West than for the Far West. Genetic clusters are named where they overlap with discrete geographic populations. Abbreviations are CANN = California nonnative, SV = Sacramento

Valley, WAC = Washington Cascades, ORC = Oregon Cascades, LAS = Lassen Cascades, SN = Sierra Nevada.

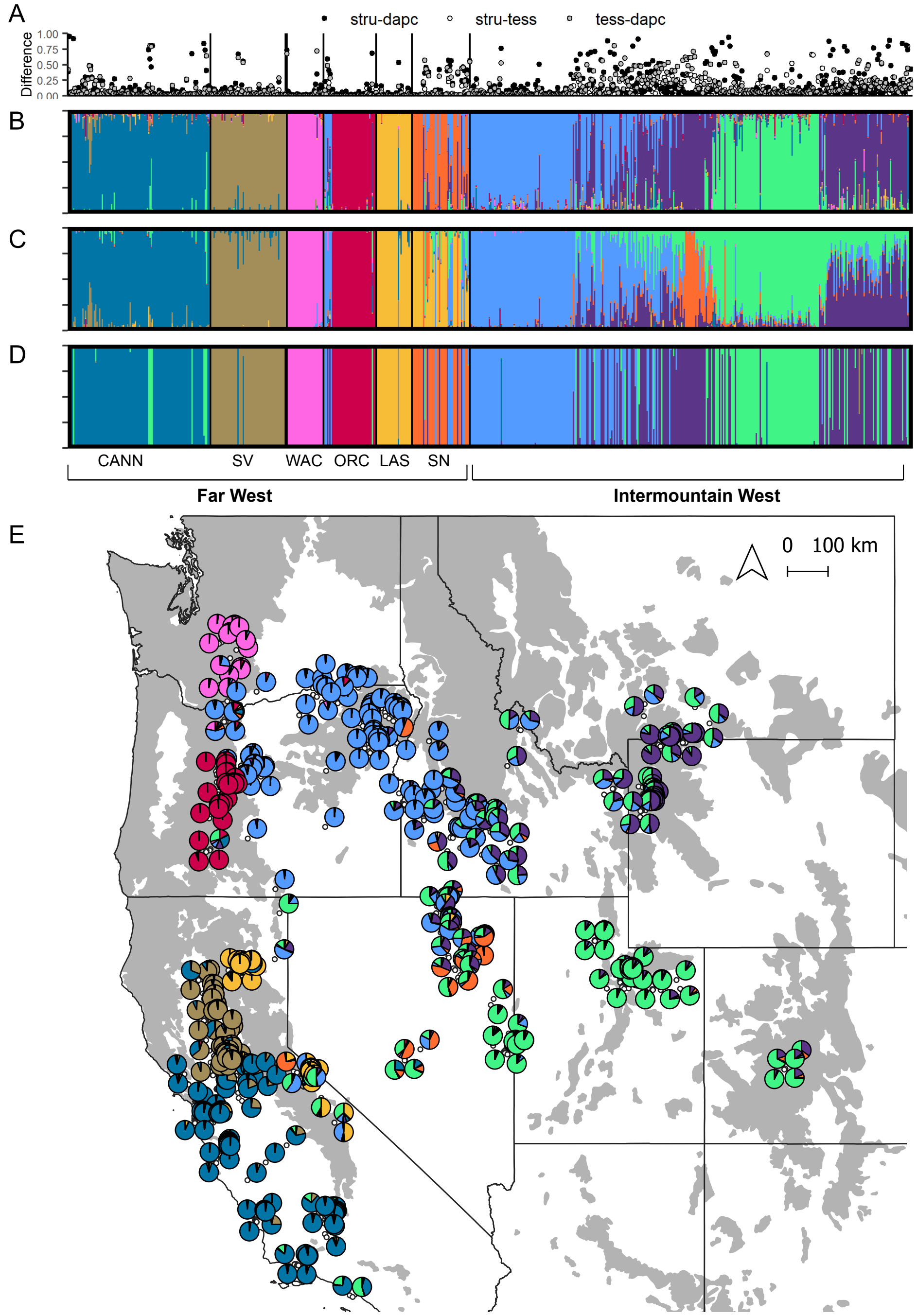

Figure S3. Genetic analysis of 642 red foxes in the western U.S., according to 31 autosomal microsatellites, at K = 9 genetic clusters. (A) Pairwise differences between individual ancestry proportions (q) determined by different algorithms, bar plots of clustering solutions from (B) Structure, (C) Tess, and (D) DAPC, and (E) pie charts showing the geographic distribution of TESS clusters. Solutions are broadly similar among methods, but there are more discrepancies in q values for individuals from the Intermountain West than for the Far West. Genetic clusters are named where they overlap with discrete geographic populations. Abbreviations are CANN = California nonnative, SV = Sacramento

Valley, WAC = Washington Cascades, ORC = Oregon Cascades, LAS = Lassen Cascades, SN = Sierra Nevada.

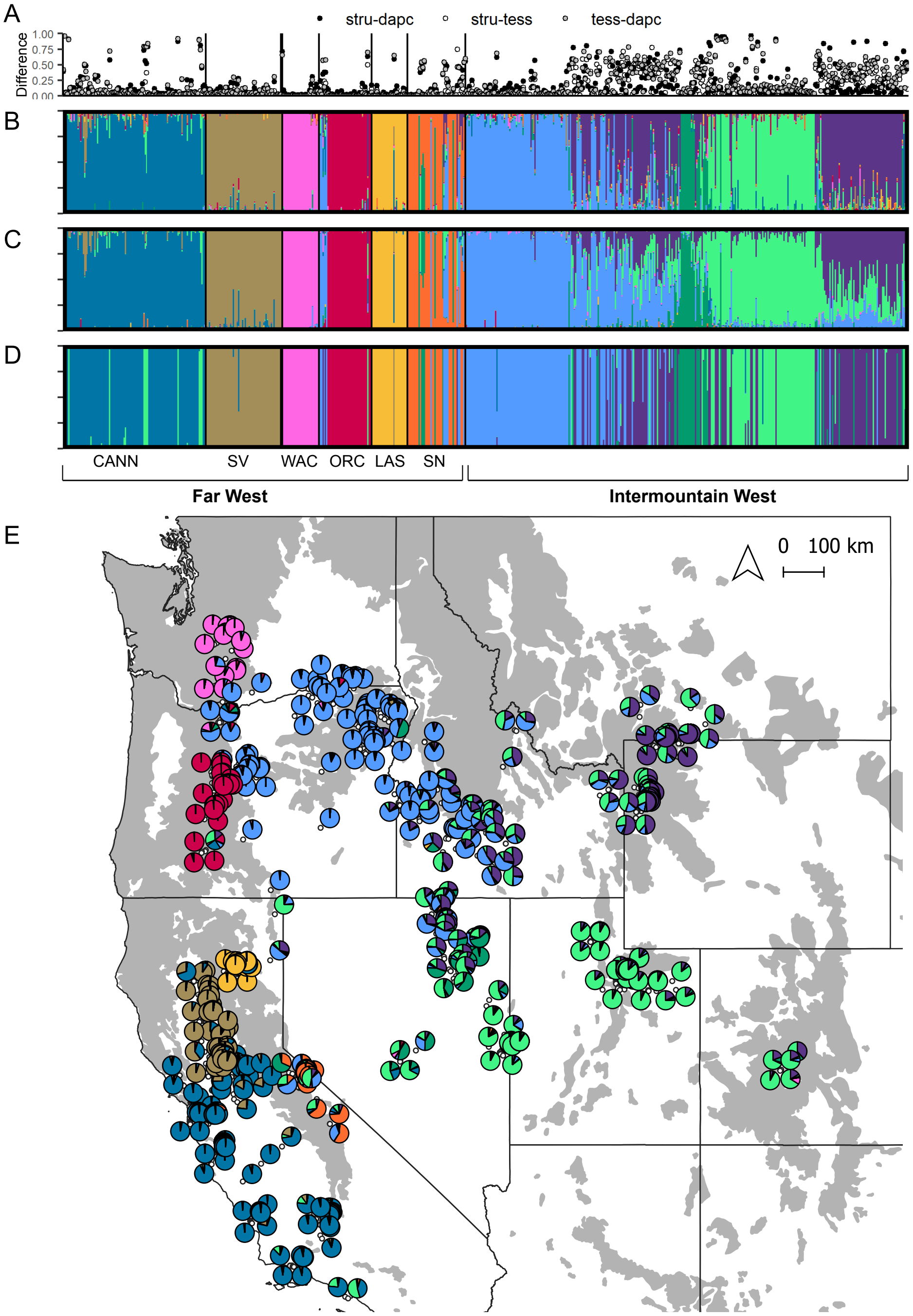

Figure S4. Genetic analysis of 642 red foxes in the western U.S., according to 31 autosomal microsatellites, at K = 10 genetic clusters. (A) Pairwise differences between individual individual ancestry proportions (q) determined by different algorithms, bar plots of clustering solutions from (B) Structure, (C) Tess, and (D) DAPC, and (E) pie charts showing the geographic distribution of TESS clusters. Solutions are broadly similar among methods, but there are more discrepancies in q values for individuals from the Intermountain West than for the Far West. Genetic clusters are named where they overlap with discrete geographic populations. Abbreviations are CANN = California nonnative, SV =

Sacramento Valley, WAC = Washington Cascades, ORC = Oregon Cascades, LAS = Lassen Cascades, SN = Sierra Nevada.

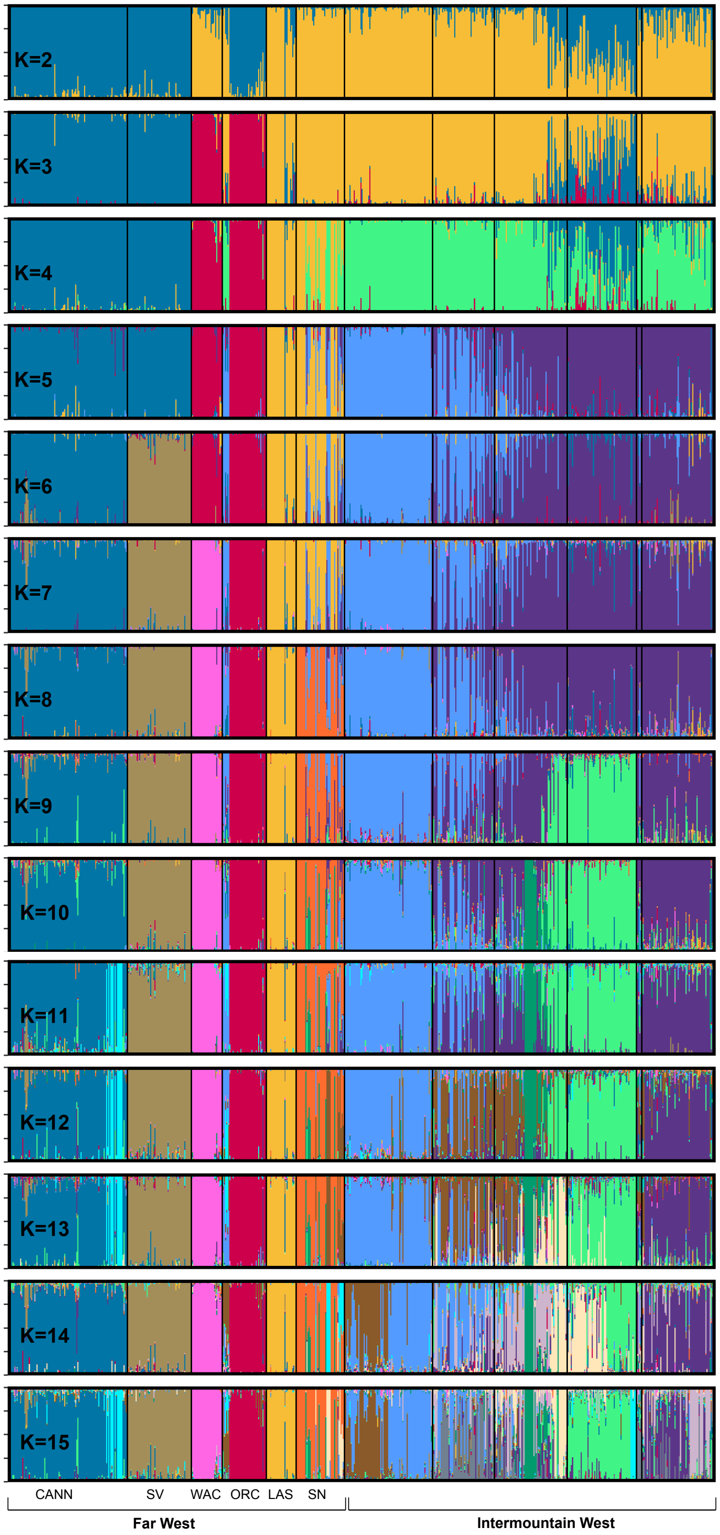

Figure S5. Genetic analysis of 642 red fox microsatellite genotypes in the western U.S. according to Structure at K = 2–15 genetic clusters. Cluster membership is strong and stable for red foxes in the Far West after K=8. Genetic clusters are named where they overlap with discrete geographic populations.

Cluster abbreviations are CANN = California nonnative, SV = Sacramento Valley, WAC = Washington Cascades, ORC = Oregon Cascades, LAS = Lassen Cascades, SN = Sierra Nevada.

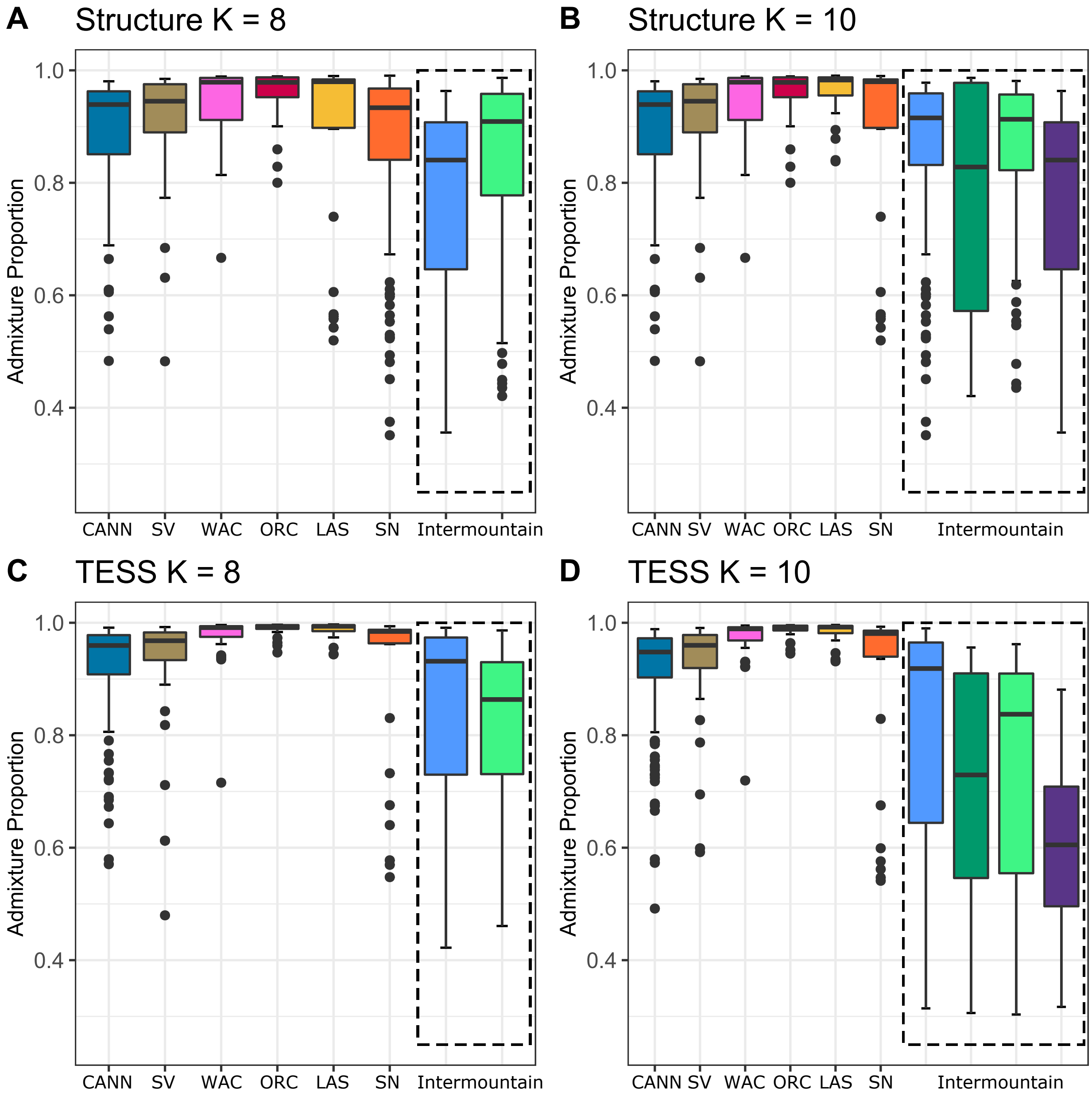

Figure S6. Distribution of admixture proportions (q values) for each genetic cluster at K=8 from Structure (A, B) and TESS (C, D). The q values for red foxes from the Intermountain West (dashed box) show greater variation and lower averages than those from the Far West, whether at K=8 (A, C), or K=10 (B, D). Cluster abbreviations are CANN = California nonnative, SV = Sacramento Valley, WAC = Washington Cascades, ORC = Oregon Cascades, LAS = Lassen Cascades, SN = Sierra Nevada.

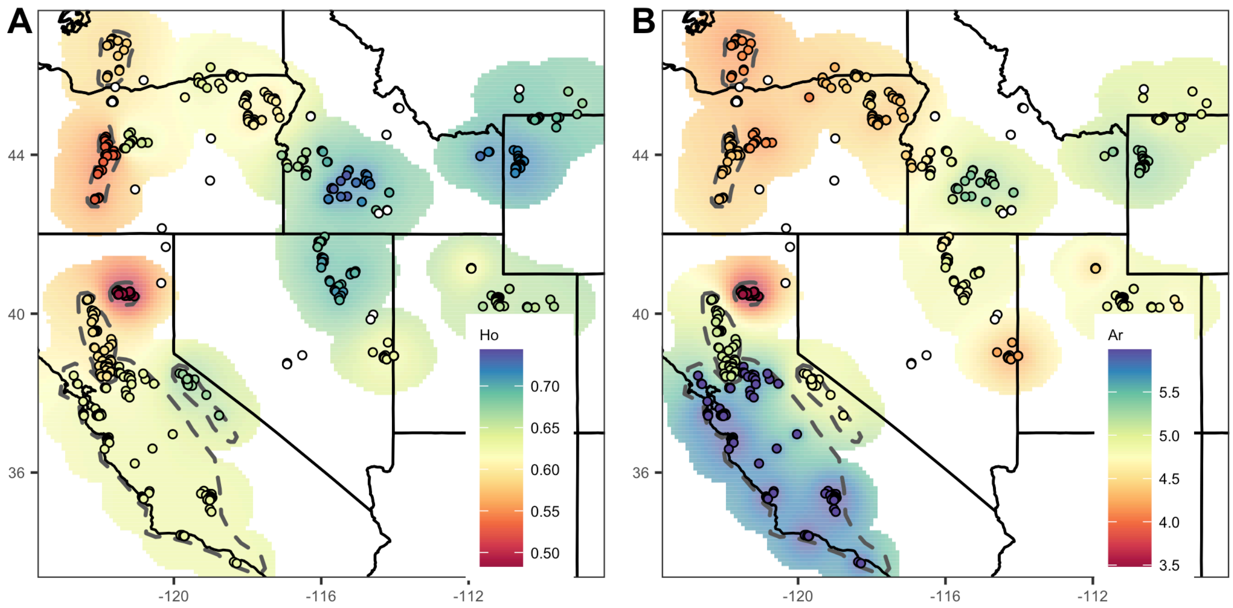

Figure S7. Spatially interpolated metrics of genetic diversity including (A) observed heterozygosity (Ho), (B) and allelic richness (Ar). Diversity metrics were calculated for populations categorized as discrete according to spatial delineation of genetic clusters (dotted lines) and for all other populations using an overlapping neighborhood approach. White circles indicate neighborhoods with <10 samples for which estimations were not attempted.

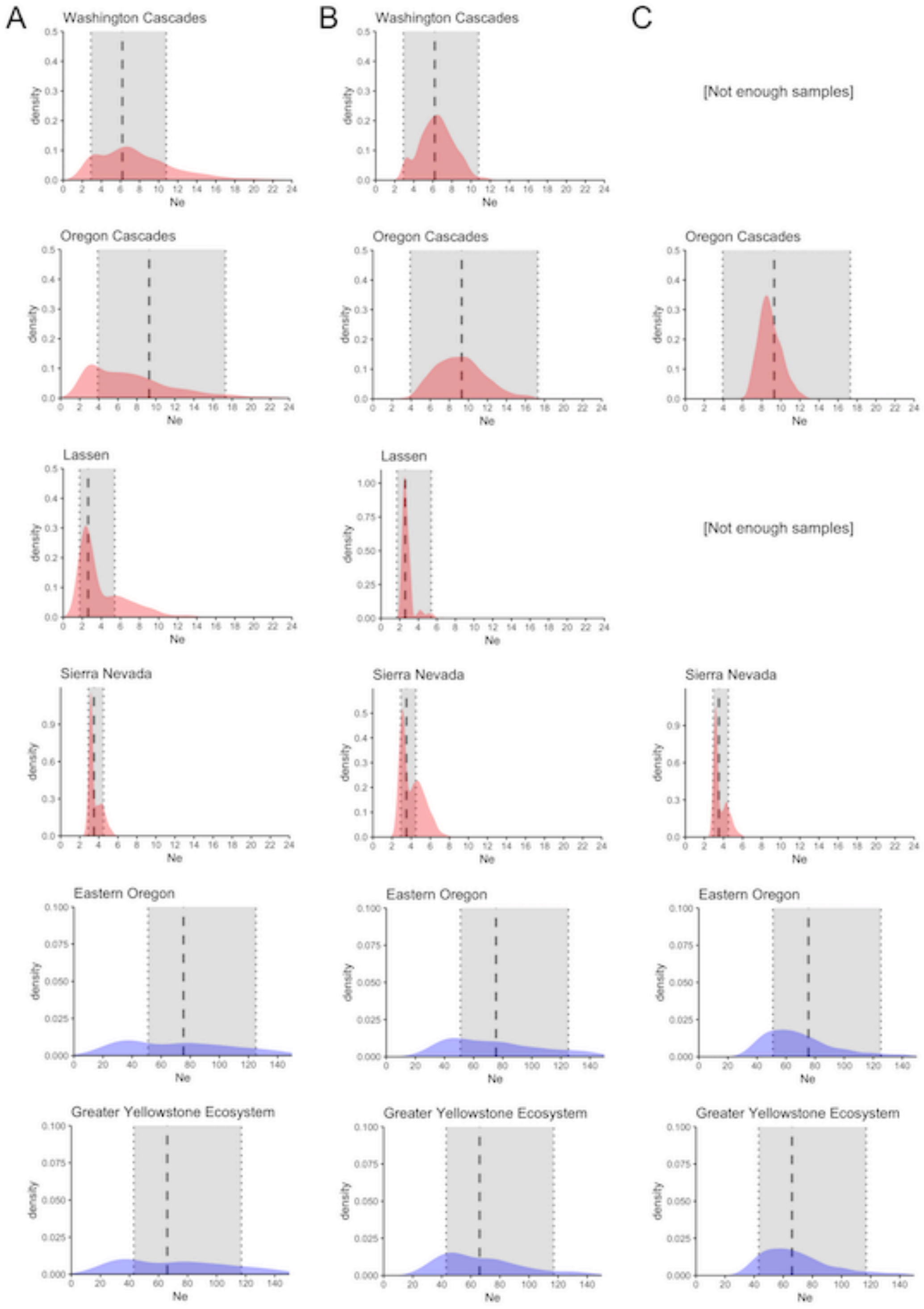

Figure S8. Sensitivity analysis of effective population sizes (N_e) estimates to sample size and underlying population structure. For each “discrete” population of the Pacific mountains (red) or geographic area of the Intermountain West where red foxes are distributed more continuously (blue), Ne was computed 1000 times by randomly subsampling without replacement (A) 10 genotypes, (B) 20 genotypes, and (C) 30 genotypes. The distribution of estimates obtained from subsampling is compared to the point estimate and

confidence intervals computed from the total samples in the region (dashed line and gray shading, respectively).

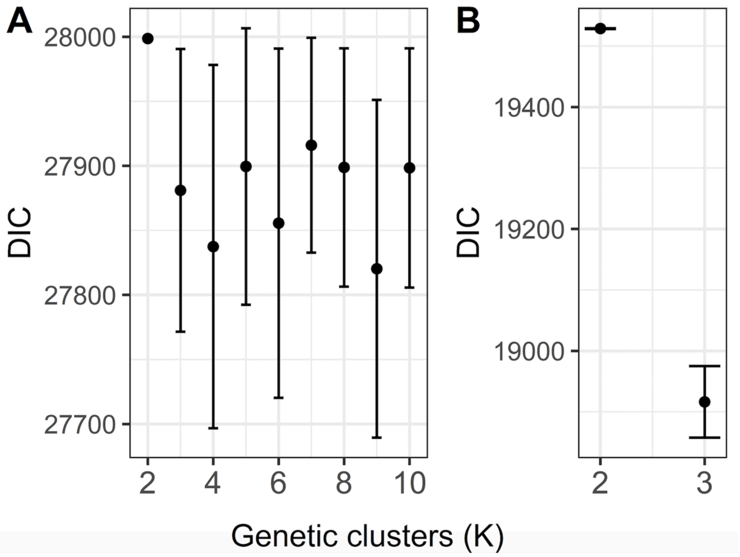

Figure S9. The DIC scores for bayseian clustering analyses using Tess, for 120 red fox samples in Oregon and southern Washington based on no-admixture models (A) and (B) admixture models. Results indicate highest performance at 3 clusters.
